# Supplementary material for: Novel DNA Aptamers that Bind to Mutant Huntingtin and Modify Its Activity
Source: Mol Ther Nucleic Acids. 2018 Mar 16;11:416–28. doi: 10.1016/j.omtn.2018.03.008 (PMC5992459; doi:10.1016/j.omtn.2018.03.008)
Supplement: Document S1. Figures S1–S8 and Tables S1–S4 [file mmc1.pdf]

OMTN, Volume *11*

Supplemental Information

Novel DNA Aptamers that Bind to Mutant Huntingtin and Modify Its Activity

Baehyun Shin, Roy Jung, Hyejin Oh, Gwen E. Owens, Hyeongseok Lee, Seung Kwak, Ramee Lee, Susan L. Cotman, Jong-Min Lee, Marcy E. MacDonald, Ji-Joon Song, Ravi Vijayvargia, and Ihn Sik Seong

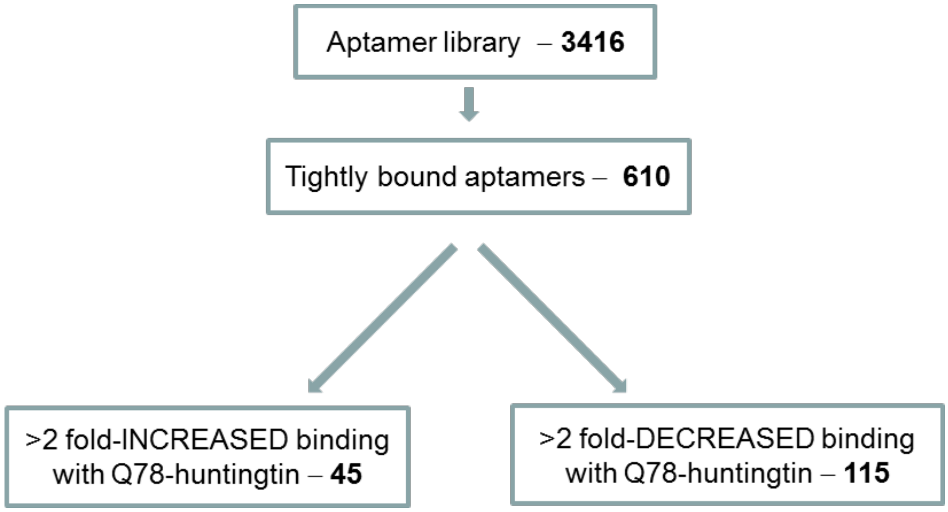

Figure S1. Experimental procedures.

Strategy for screening of Q78-huntingtin specific DNA aptamers.

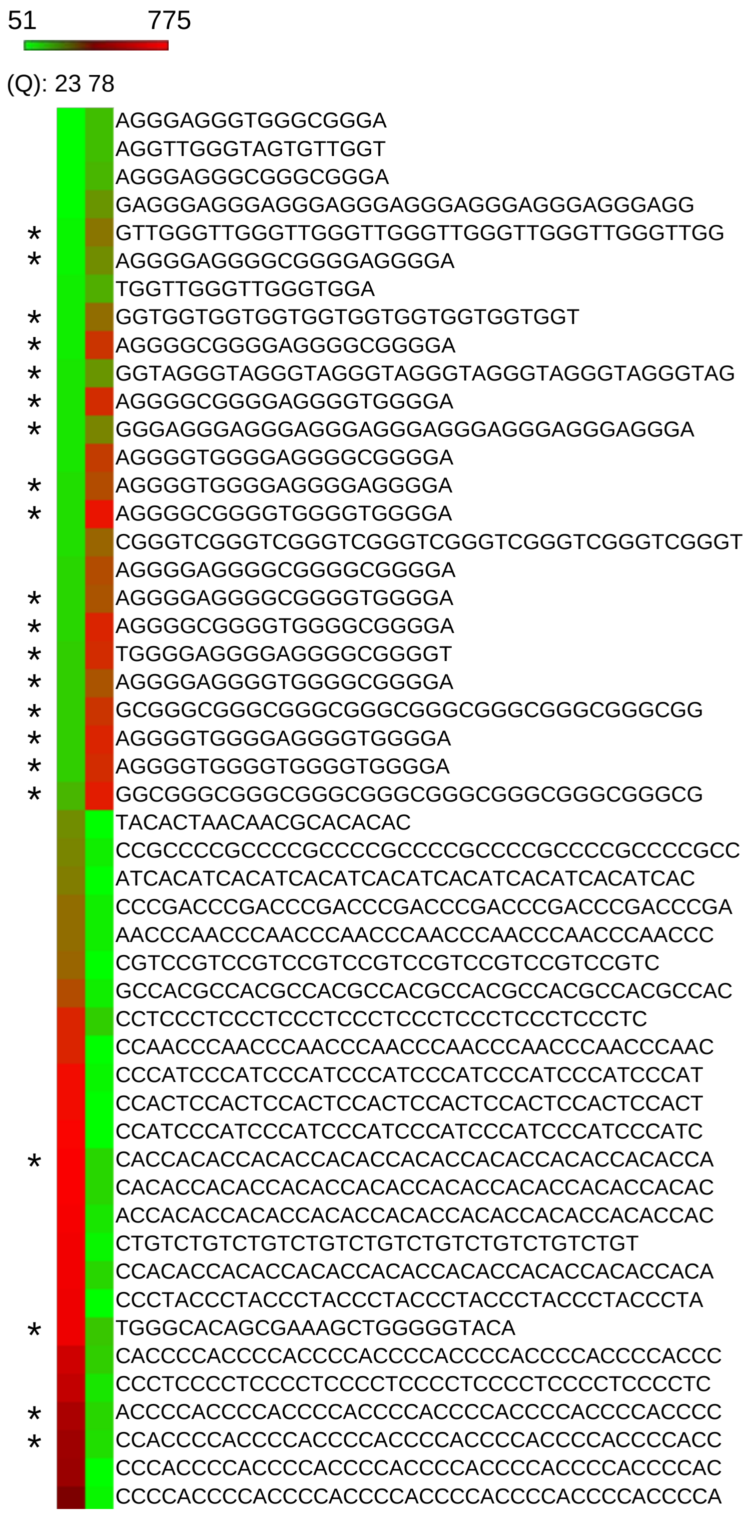

Figure S2. Selected DNA aptamers from microarray.

Heat map of Q23/Q78-huntingtin-preferring DNA aptamers and their sequences. High affinity is shown in shades of red while low affinity is shown in shades of green. Asterisks indicate 21 representative aptamers chosen for an ELISA assay.

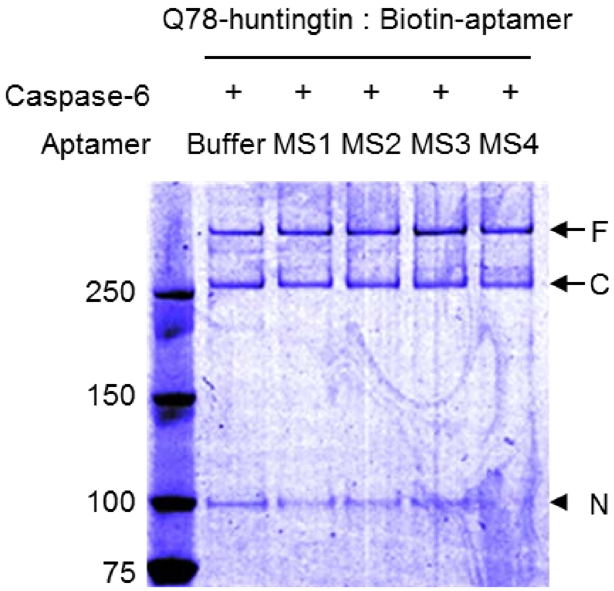

Figure S3. Aptamer binding does not alter cleavage of Q78-huntingtin by caspase-6.

Biotinylated DNA aptamer (MS1, 2, 3, 4)-bound Q78-huntingtin was cleaved by caspase-6. The cleavage products were separated by SDS-PAGE and detected by Coomassie staining. (F: full-length, C: carboxyl-terminal fragment, N: amino-terminal fragment)

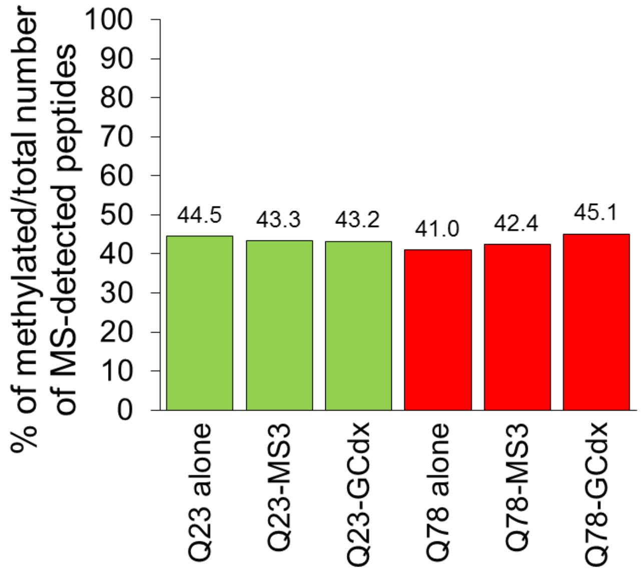

Figure S4. Identification of MS3 aptamer binding site.

Bar graph showing the percentage of methylated peptides within total MS-detected peptides from huntingtin alone or huntingtin-aptamer complexes indicating the overall methylation reaction of all samples was performed identically.

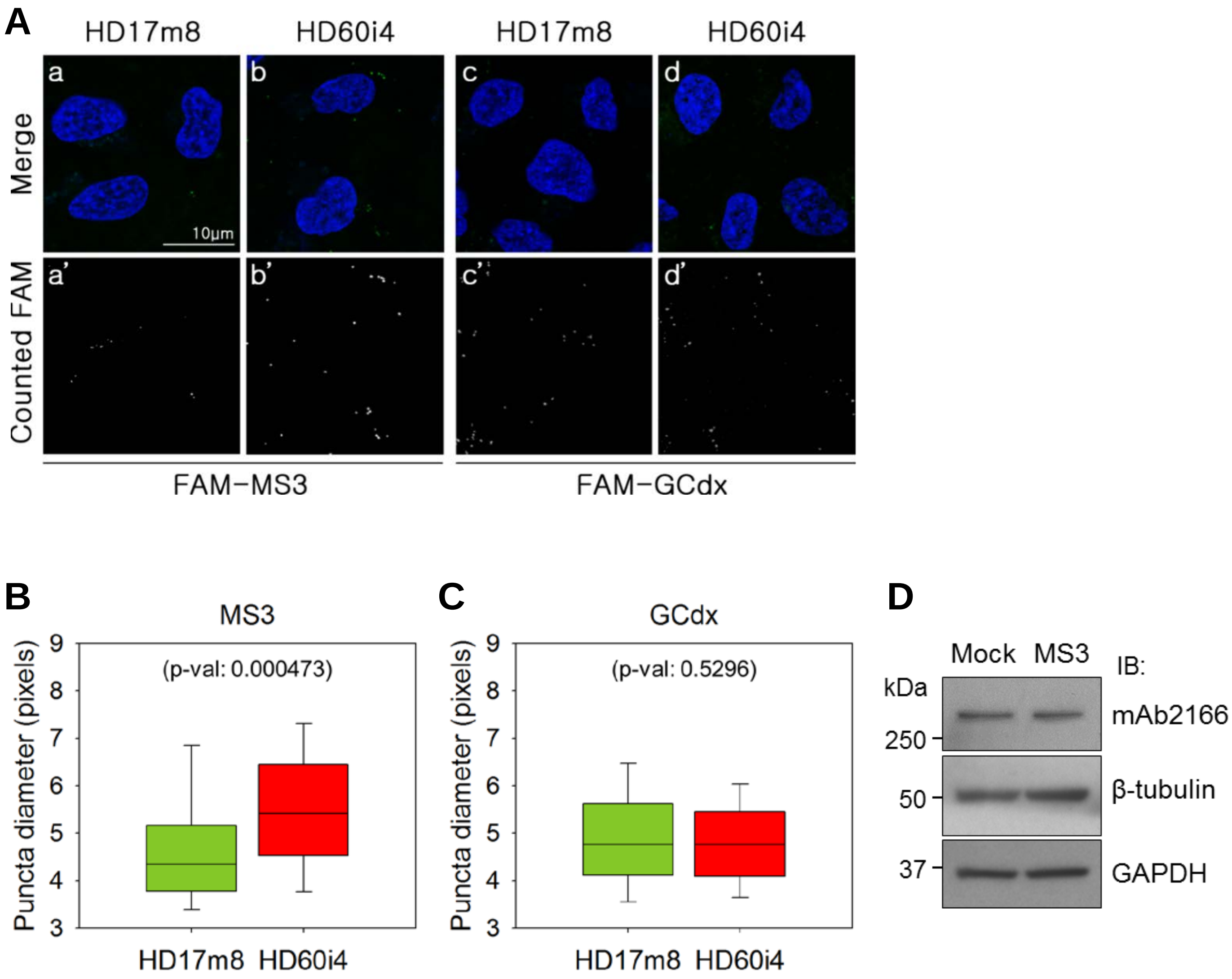

Figure S5. The accumulation of transfected FAM-MS3 aptamers was significantly increased in HD60i4 as compared to HD17m8.

(**A**) The merged fluorescence images of HD17m8 and HD60i4 NPCs (panel *a-d*), showing the Hoechst 33342 (blue) nuclei and transfected 6-FAM (green)-conjugated MS3 and GCdx signal, illustrating that the diameter of FAM-MS3 puncta were significantly enlarged in HD60i4 NPCs compared to HD17m8, whereas FAM-GCdx puncta showed similar fluorescence intensities in both HD17m8 and HD60i4 hNPC. Scale bar, 10 μm. (**B, C**) The diameter of each FAM-MS3 (B) and FAM-GCdx (C) puncta signal in HD17m8 and HD60i4 hNPC were displayed by vertical box plot. Mean puncta diameter was measured in 12 images per condition. Error bars represent S.E.M. *P*-values determined by a two-sided unpaired Student’s *t*-test. (**D**) Immunoblot showing the amount of endogenous huntingtin without or with MS3 aptamer transfection in HD60i4 NPC by probing with anti-huntingtin antibody (mAb2166). β-tubulin and GAPDH, loading controls.

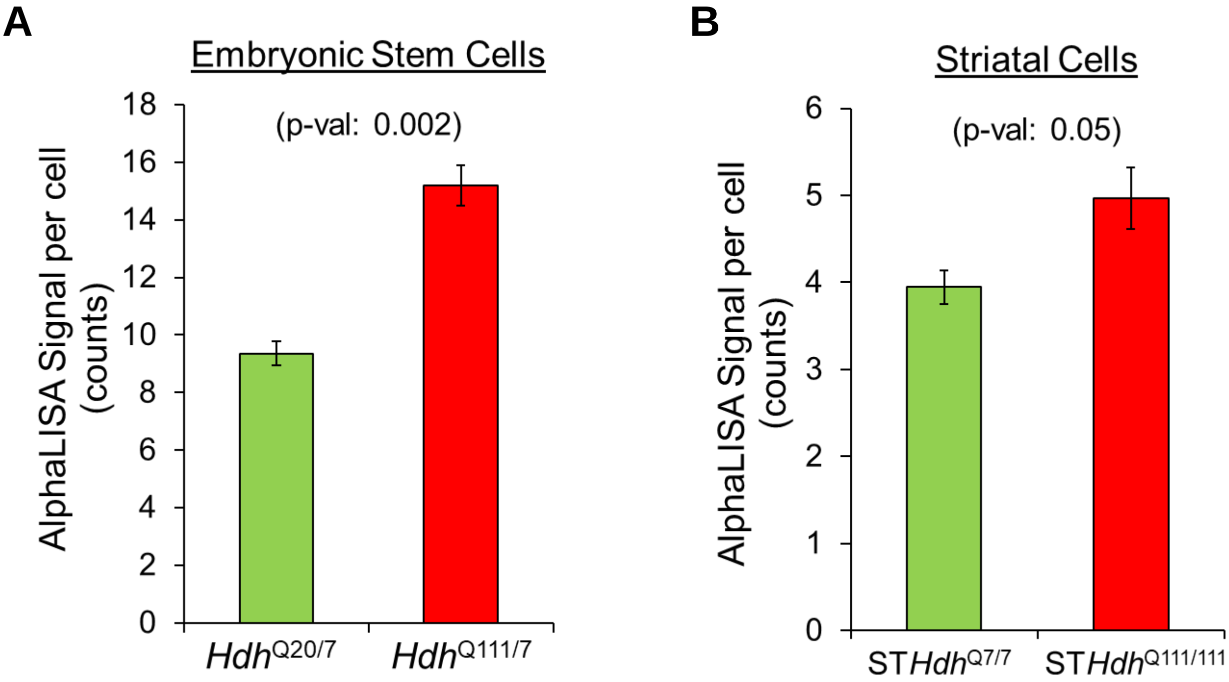

Figure S6. The endogenous levels for H3K27me3 in HD cellular models were significantly increased compared to wild-type.

(**A**) The AlphaLISA signal of H3K27me3 was significantly elevated in mouse embryonic stem cells with CAG 111/7 (*Hdh*$^{Q111/7}$) compare to cells with CAG 20/7 (*Hdh*$^{Q20/7}$). (**B**) The AlphaLISA signal of H3K27me3 was significantly elevated in *Hdh* CAG knock-in mouse striatal cell line with CAG 111/111 (ST*Hdh*$^{Q111/111}$) compare to cells with CAG 7/7 (ST*Hdh*$^{Q7/7}$). Mean of AlphaLISA signal was obtained from three independent experiments and the error bars represent S.E.M. *P*-values determined by a two-sided unpaired Student's *t*-test.

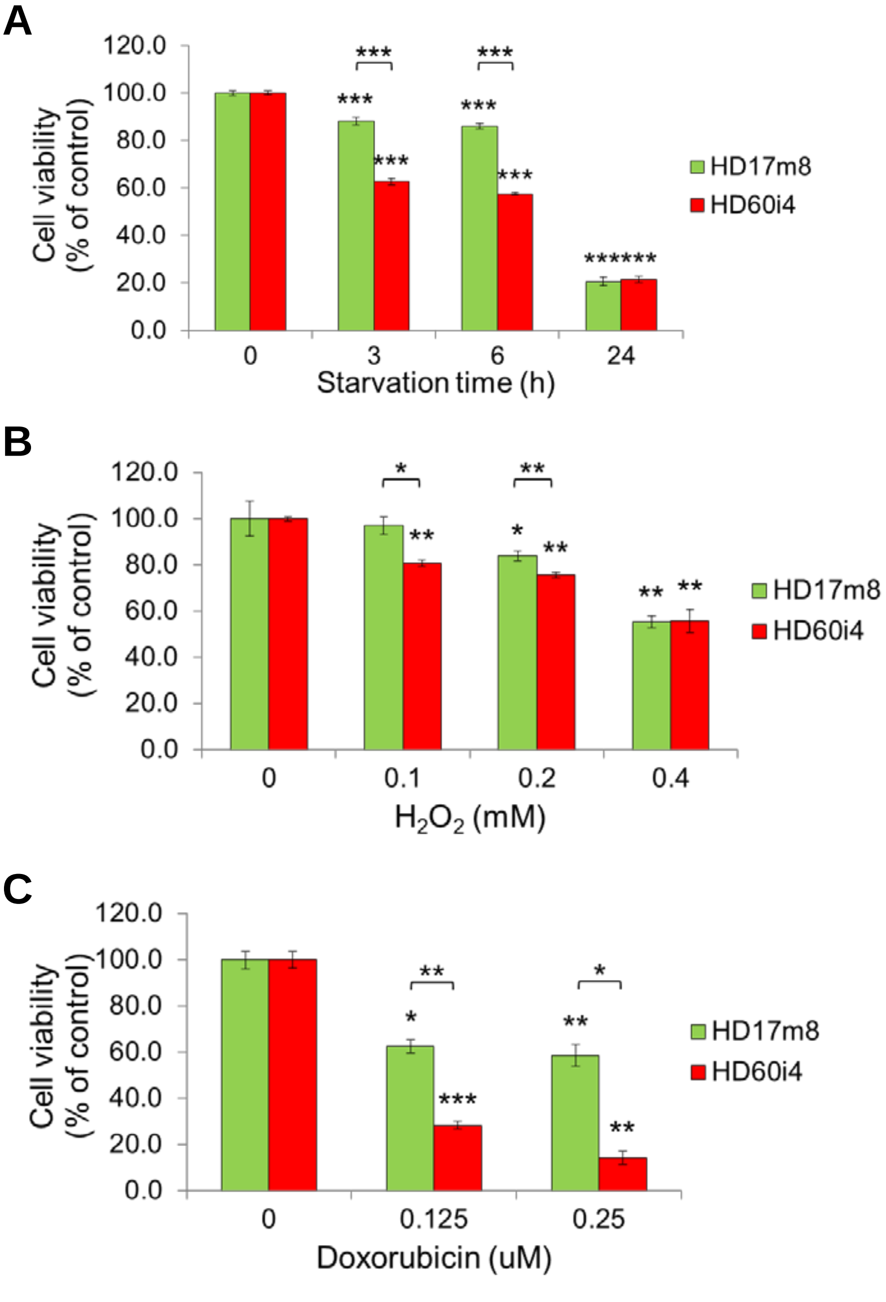

Figure S7. Measurement of cell viabilities in HD17m8 and HD60i4 NPCs under various cellular stress.

(**A**) Time course analyses of cell viability for NPCs starved in total starvation medium (HBSS) for 0, 3, 6 or 24 h. (**B**) Cell viability in NPCs cultured in the absence or presence of H_2O_2 (0.1, 0.2 or 0.4 mM) for 6 h. (**C**) Cell viability in NPCs cultured in the absence or presence of doxorubicin (0.125 or 0.25 µM) for 24 h. Values are presented as the mean of three independent experiments. Error bars represent S.E.M. **P* < 0.05, ***P* < 0.01, ****P* < 0.001 (two-sided unpaired Student's t-test).

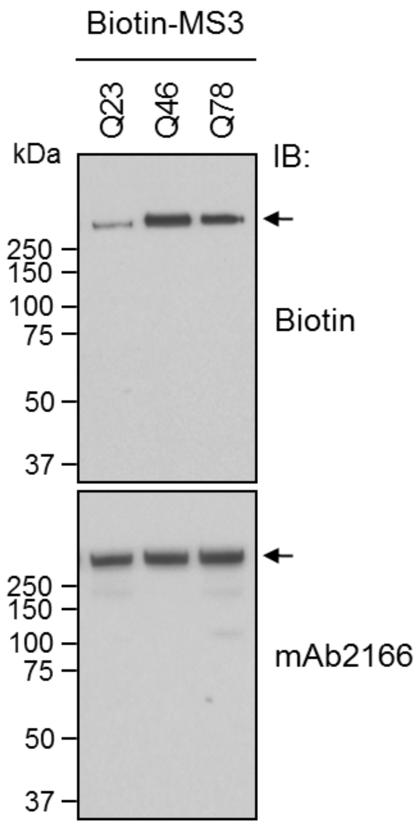

Figure S8. *In vitro* binding assay of MS3 aptamer with Q23-, Q46-, Q78-huntingtin. Representative immunoblot showing purified Q23-, Q46- and Q78-huntingtin bound with biotinylated MS3 aptamer by probing with an anti-biotin antibody (Biotin) and an anti-huntingtin antibody (mAb2166). The location of full-length huntingtin is indicated by the arrow in both immunoblots. The experiment was repeated three times.

Aptamer	DNA Sequence (5' to 3')	Microarray (Mean O.D.)		ELISA (Mean O.D.)	
		Q23	Q78	Q23	Q78
MS1	AGGGGTGGGGAGGGGTGGGGA	123.87	356.95	0.179	0.394
MS2	AGGGGTGGGGAGGGGAGGGGA	98.36	302.70	0.306	0.605
MS3	GGGAGGGAGGGAGGGAGGGAGGGAGGGAGGGAGGGA	88.53	227.67	0.347	0.730
MS4	TGGGGAGGGGAGGGGCGGGGT	118.40	363.94	0.152	0.388
MS5	AGGGGAGGGGCGGGGTGGGGA	109.69	295.69	0.228	0.327
MS6	AGGGGAGGGGTGGGGCGGGGA	122.09	296.94	0.078	0.241
MS7	AGGGGCGGGGAGGGGTGGGGA	87.54	347.76	0.115	0.262
MS8	AGGGGCGGGGTGGGGCGGGGA	110.22	354.84	0.142	0.244
MS9	AGGGGCGGGGAGGGGCGGGGA	83.37	333.58	0.066	0.184
MS10	AGGGGAGGGGCGGGGAGGGGA	71.63	211.30	0.125	0.174
MS11	AGGGGTGGGGTGGGGTGGGGA	125.42	348.68	0.031	0.069
MS12	GGTGGTGGTGGTGGTGGTGGTGGTGGTGGT	82.49	260.67	0.103	0.185
MS13	GCGGGCGGGCGGGCGGGCGGGCGGGCGGGCGGGCGG	122.77	338.75	0.291	0.342
MS14	GGCGGGCGGGCGGGCGGGCGGGCGGGCGGGCGGGCG	155.39	366.39	0.303	0.298
MS15	GGTAGGGTAGGGTAGGGTAGGGTAGGGTAGGGTAGGGTAG	85.84	199.43	0.019	0.036
MS16	GTTGGGTTGGGTTGGGTTGGGTTGGGTTGGGTTGGGTTGG	64.26	252.61	0.140	0.316
MS17	AGGGGCGGGGTGGGGTGGGGA	99.25	378.31	0.244	0.363
WS1	CACCACACCACACCACACCACACCACACCACACCACACCA	423.64	118.00	0.095	0.159
WS2	TGGGCACAGCGAAAGCTGGGGGTACA	456.74	129.55	0.080	0.086
WS3	CCACCCCACCCCACCCCACCCCACCCCACCCCACCCCACC	552.47	123.68	0.039	0.065
WS4	ACCCCACCCCACCCCACCCCACCCCACCCCACCCCACCCC	651.73	110.78	0.048	0.120

Table S1. Table showing microarray and ELISA signal of the selected DNA aptamers with Q23/Q78-huntingtin. All 17 DNA aptamers that preferentially bound to Q78-huntingtin in the microarray experiment were consistent in ELISA assay while 4 DNA aptamers preferring Q23-huntingtin in the microarray experiment failed to be reproduced and, instead, showed low affinities to both Q23- and Q78-huntingtin in ELISA assay.

Aptamer	DNA Sequence (5' to 3')
MS1	AGGGGTGGGGAGGGGTGGGGA
MS2	AGGGGTGGGGAGGGGAGGGGA
MS3	GGGAGGGAGGGAGGGAGGGAGGGAGGGAGGGAGGGA
MS4	TGGGGAGGGGAGGGGCGGGGT
c-myc	TGAGGGTGGGTAGGGTGGGTAA
45Ag	GGGTTAGGGTTAGGGTTAGGGTTAGGGTTAGGGTTAGGGTTAGGG
ds26	CAATCGGATCGAATTCGATCCGATTG
GCdx	GCGCGCGCTTTTGCGCGCGC

Table S2. DNA sequences used for the Thioflavin T (ThT) fluorescence assay.

A

Position	Number of methylated peptides		
	Q23 alone	Q23-MS3	Q23-GCdx
K125	5	3	4
K236	5	5	8
K251	6	8	11
K255	5	4	5
K262	4	6	4
K332	10	12	9
K337	7	4	4
K410	8	6	26
K473	5	5	4
K648	10	10	8
K664	8	24	16
R667	7	11	4
K669	23	19	25
K700	13	13	17
K738	3	2	1
K904	3	6	6
K943	9	14	12
K965	6	10	9
K1105	5	4	5
K1168	7	6	4
K1225	37	37	32
K1246	4	1	0
K1264	9	10	10
K1319	5	5	5
K1339	19	20	18
K1398	4	5	10
K1417	79	88	84
K1433	48	56	54
K1479	4	5	5
K1514	10	8	7
K1521	12	10	10
K1763	5	3	2
K1861	18	22	16
K2163	7	11	13
K2236	6	6	8
K2425	5	11	7
K2445	4	3	6
K2539	34	18	34
K2548	6	10	11
K2566	7	6	3
K2598	3	2	5
K2759	9	5	8
K2802	3	1	0
K2903	4	4	5
K2932	46	34	35
K2934	46	34	35
K2969	5	4	4
K3020	11	10	2
K3140	76	80	45

B

Position	Number of methylated peptides		
	Q78 alone	Q78-MS3	Q78-GCdx
K125	4	4	6
K236	8	9	11
K251	9	7	10
K255	5	5	4
K262	6	6	6
K332	13	13	15
K337	6	5	7
K410	25	43	52
K473	6	4	5
K648	11	13	10
K664	40	14	20
R667	20	4	10
K669	16	20	21
K700	13	12	14
K888	3	2	3
K904	6	2	6
K943	15	17	15
K965	12	14	13
K1062	3	4	3
K1105	4	4	6
K1168	7	6	6
K1225	41	27	37
K1264	13	11	14
K1319	5	5	5
K1339	19	30	22
R1343	4	1	2
K1398	9	9	10
K1417	86	84	90
K1433	48	56	58
K1479	7	7	8
K1514	10	8	9
K1521	12	9	11
K1570	3	2	2
K1763	5	3	5
# K1861	5	31	46
K2163	7	9	15
K2236	5	9	10
K2425	7	8	9
K2445	7	8	9
K2449	6	0	5
K2539	20	17	42
K2548	10	8	8
K2566	3	8	7
R2567	5	6	3
K2598	3	3	3
K2615	5	0	2
K2759	6	6	9
K2903	3	4	6
K2932	52	12	66
K2934	52	12	66
K2969	4	4	5
K3020	3	2	5
K3084	3	3	3
K3140	35	45	87

Table S3. (**A, B**) Table showing the number of methylated peptides detected by LC/MS/MS from huntingtin alone or huntingtin (Q23/Q78)-aptamer complexes. Of note, there were a few sites (e.g., #K1861 in Q78-huntingtin) showing a highly negative score due to a high B/A (aptamer bound huntingtin/unbound huntingtin) value. This may have resulted from the incidental activation of the SLM reaction by the presence of aptamer on the surface near the site, suggested by the observation that, while GCdx exhibited no specific binding to huntingtin, methylation at that site in the presence of GCdx was relatively high compared to Q78 alone (46 versus 5 methylated peptides).

Category	Parameter	Description
Assay	Type of assay	*In vitro* Microarray chip
	Target	Human full-length huntingtin with Q23 and Q78 P42858 (HD_HUMAN)
	Primary measurement	Detection of fluorescence signal from huntingtin protein that bound tightly with single-strand DNA aptamer
	Key reagents	Alexa fluorophore labeled purified full-length huntingtin proteins
	Assay protocol	See the methods section titled in 'Aptamer screening using the recombinant purified full-length huntingtin'
	Additional comments	
Library	Library size	3,416
	Library composition	3,416 single-stranded DNA aptamer probes
	Source	Single-strand DNA format collected from literature or database
	Additional comments	
Screen	Format	Microarray
	Concentration(s) tested	1μM (200 μl)
	Plate controls	Multiple quality control probes are included on each chip
	Reagent/ compound dispensing system	Manual addition of proteins
	Detection instrument and software	Axon GenePix 4000B Microarray Scanner
	Assay validation/QC	Multiple quality control probes are included on each chip
	Correction factors	Background is calculated from the median of 5% to 25% of low intensity cells. BKG0 and blank cells are excluded for the background calculation
	Normalization	No Normalization
	Additional comments	
Post-HTS analysis	Hit criteria	Preferential binding to target Huntingtin proteins
	Additional assay(s)	17 screened DNA aptamers were further validated by ELISA assay
	Confirmation of hit purity and structure	17 Hits (DNA aptamers) were re-synthesized and their specificity to huntingtin was confirmed by ELISA
	Additional comments	

Table S4. High-throughput screening of DNA aptamers preferentially bind to mutant huntingtin.
