# Supplementary material for: Novel DNA Aptamers that Bind to Mutant Huntingtin and Modify Its Activity
Source: Mol Ther Nucleic Acids. 2018 Mar 16;11:416–28. doi: 10.1016/j.omtn.2018.03.008 (PMC5992459; doi:10.1016/j.omtn.2018.03.008)
Supplement: Document S2. Article plus Supplemental Information [file mmc2.pdf]

Molecular Therapy
Nucleic Acids
Original Article

Novel DNA Aptamers that Bind to Mutant Huntingtin and Modify Its Activity

Baehyun Shin,[1,2] Roy Jung,[1,2] Hyejin Oh,[1,2] Gwen E. Owens,[3] Hyeongseok Lee,[4] Seung Kwak,[5] Ramee Lee,[5] Susan L. Cotman,[1,2] Jong-Min Lee,[1,2] Marcy E. MacDonald,[1,2] Ji-Joon Song,[4] Ravi Vijayvargia,[1,2,6] and Ihn Sik Seong[1,2]

[1]Center for Genomic Medicine, Massachusetts General Hospital, Boston, MA 02114, USA; [2]Department of Neurology, Harvard Medical School, Boston, MA 02114, USA; [3]Division of Biology and Biological Engineering, California Institute of Technology, Pasadena, CA 91125, USA; [4]Department of Biological Sciences, KAIST Institute for the BioCentury, Center for Cancer Metastasis, Korea Advanced Institute of Science and Technology (KAIST), Daejeon 34141, Republic of Korea; [5]CHDI Foundation, Princeton, NJ 08540, USA

The CAG repeat expansion that elongates the polyglutamine tract in huntingtin is the root genetic cause of Huntington's disease (HD), a debilitating neurodegenerative disorder. This seemingly slight change to the primary amino acid sequence alters the physical structure of the mutant protein and alters its activity. We have identified a set of G-quadruplex-forming DNA aptamers (MS1, MS2, MS3, MS4) that bind mutant huntingtin proximal to lysines K2932/K2934 in the C-terminal CTD-II domain. Aptamer binding to mutant huntingtin abrogated the enhanced polycomb repressive complex 2 (PRC2) stimulatory activity conferred by the expanded polyglutamine tract. In HD, but not normal, neuronal progenitor cells (NPCs), MS3 aptamer co-localized with endogenous mutant huntingtin and was associated with significantly decreased PRC2 activity. Furthermore, MS3 transfection protected HD NPCs against starvation-dependent stress with increased ATP. Therefore, DNA aptamers can preferentially target mutant huntingtin and modulate a gain of function endowed by the elongated polyglutamine segment. These mutant huntingtin binding aptamers provide novel molecular tools for delineating the effects of the HD mutation and encourage mutant huntingtin structure-based approaches to therapeutic development.

INTRODUCTION

Huntington's disease (HD) is a dominantly inherited neurodegenerative disorder with motor, cognitive, and psychiatric features caused by expanded *HTT* CAG trinucleotide repeats that extend a polyglutamine tract in the amino terminus of huntingtin.[1] In general, the longer the *HTT* CAG repeat expansion, the earlier the age at onset of the clinical HD features.[2,3] This inverse correlation between CAG length and age at onset is also observed for CAG repeat expansion mutations that cause other clinically distinct inherited neurodegenerative disorders such as spinobulbar muscular atrophy and spinocerebellar ataxias, with the mutation in each case extending a polyglutamine tract in a different protein.[4,5] The distinct protein contexts, in each of these disorders, strongly suggest that the mechanism that initiates the disease cascade leading to the clinical symptoms of HD likely entails some property conferred by the expanded polyglutamine tract on mutant huntingtin.

Huntingtin is a flexible ~3,144-aa HEAT/HEAT-like (Huntingtin, Elongation factor 3, protein phosphatase 2A, Target of rapamycin 1) α-helical solenoid protein. Huntingtin likely serves as a scaffold for multi-member complexes.[6–8] Using purified human recombinant huntingtins, with different polyglutamine segment lengths, we have employed a variety of biochemical and biophysical methods to delineate the domain structures of normal and mutant huntingtin.[8] Huntingtins, regardless of polyglutamine length, possess a major hinge region that delineates a 150-kDa amino-terminal arm, comprising two domains (NTD-I and NTD-II), and a 200-kDa C-terminal arm, with three domains (CTD-I, uncrosslinked domain [UCD], and CTD-II).[8] Consistent with a continuous α-helical structure, formed by stacking of adjacent HEAT/HEAT-like repeats, the intramolecular contacts at sites within the domains of the amino-terminal and C-terminal arms are modulated by the length of the polyglutamine segment in NTD-I.[8] In addition, extending the length of the polyglutamine tract enhances the ability of recombinant human mutant huntingtin to stimulate the basal histone H3 lysine 27 trimethylation (H3K27me3) activity of polycomb repressive complex 2 (PRC2), as measured in a cell-free chromatin-nucleosome assay.[9] This quantitative biochemical assay, which serves to monitor the effect of the polyglutamine tract length on a functional activity of huntingtin, emerged from huntingtin's critical role in regulating PRC2

Received 28 December 2017; accepted 13 March 2018;
https://doi.org/10.1016/j.omtn.2018.03.008.

[6]Present address: Department of Biochemistry, The Maharaja Sayajirao University of Baroda, Vadodara, Gujarat 390 002, India.

Correspondence: Ravi Vijayvargia, Center for Genomic Medicine, Massachusetts General Hospital, Boston, MA 02114, USA.
E-mail: ravi.vijayvargia-biochem@msubaroda.ac.in
Correspondence: Ihn Sik Seong, Center for Genomic Medicine, Massachusetts General Hospital, Boston, MA 02114, USA.
E-mail: iseong@mgh.harvard.edu

This is an open access article under the CC BY license (http://creativecommons.org/licenses/by/4.0/).

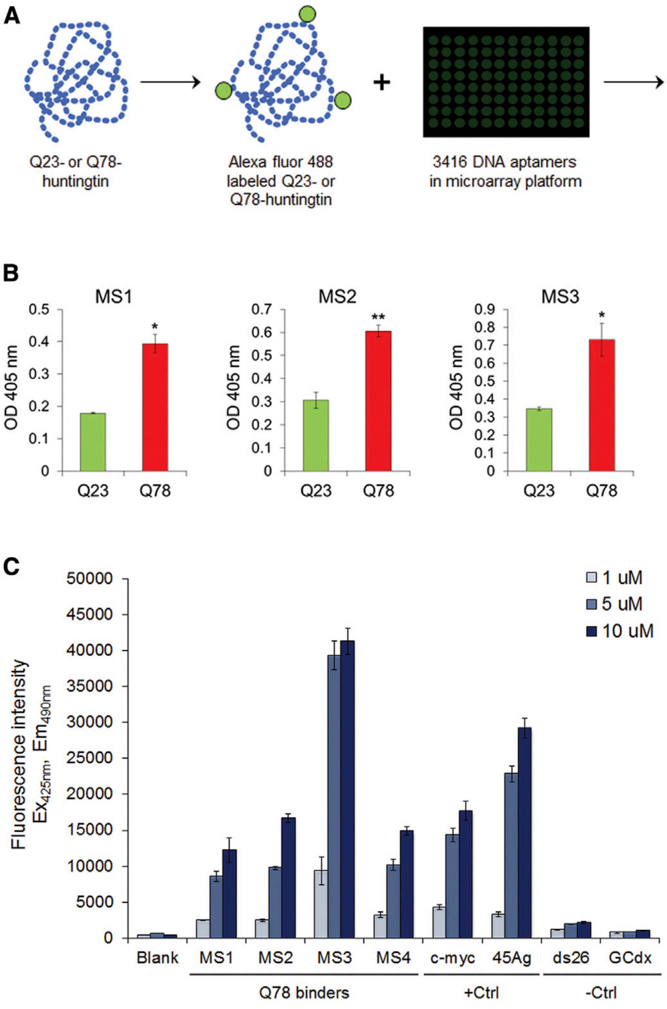

Figure 1. Screening of DNA Aptamers that Can Preferentially Bind to Q78-Huntingtin

(A) A schematic illustrating DNA aptamer screening to identify aptamers that bind preferentially to Q23- or Q78-purified human recombinant huntingtin. (B) Bar graphs presenting ELISA data show four representative aptamers that bind significantly more to the purified Q78-huntingtin (Q78) compared with Q23-huntingtin (Q23). Mean optical densities at 405 nm were measured by ELISA plate reader from three independent experiments. $*p < 0.05$; $**p < 0.01$; $***p < 0.001$ (two-sided unpaired Student's t test). (C) Thioflavin T fluorescence assay was performed with Q78-huntingtin preferring aptamers, as well as positive and negative control DNA aptamers. G-quadruplex-forming DNA oligonucleotides c-*myc* and 45Ag produced a strong positive signal, whereas ds26, which forms a DNA duplex, and GCdx, which forms a stem-loop structure, had very weak signals just above baseline. Error bars represent SEM.

deposition of the chromatin histone H3K27me3 mark in developing mouse embryos.[9]

These observations suggested to us that small molecules that preferentially bind to the altered structure of mutant huntingtin, compared with normal huntingtin, may selectively modulate the gain of function conferred on the former by its longer polyglutamine tract. DNA aptamers, which are emergent in biomedical applications for biosensing, diagnostics, and therapeutics, are single-stranded oligonucleotides that can be selected from large random sequence pools through the specific high-affinity associations of these small molecules with target proteins.[10,11] To provide an initial proof of concept for small-molecule binding as a route to directly modulate mutant huntingtin, we have screened a library of single-stranded DNA aptamers to identify those that bind preferentially to highly purified recombinant human mutant huntingtin (78-glutamine tract), but not normal huntingtin (23-glutamine tract). This strategy yielded a unique set of aptamers, binding the C terminus, which we have evaluated for their potential ability to modulate the gain of function endowed on the mutant protein by its expanded polyglutamine tract.

RESULTS

A Set of G-Quadruplex DNA Aptamers that Preferentially Bind to Mutant Huntingtin

We screened a library of DNA aptamers to select for those able to discriminate between highly purified recombinant human huntingtin with a normal polyglutamine tract of 23 residues (hereafter Q23-huntingtin) or with an expanded 78-residue polyglutamine tract (hereafter Q78-huntingtin), using a microarray platform comprising 3,416 DNA aptamers (Figure 1A). Because we expect subtle structural changes in mutant huntingtin that are correlated to polyglutamine tract size,[8] we chose Q78-huntingtin to maximize the possibility to find out mutant huntingtin-specific DNA aptamers. From the DNA aptamers that tightly bound to either huntingtin, subsets of aptamers that preferentially bound to normal or to mutant huntingtin were then chosen using a 2-fold cutoff (Figure S1). Interestingly, the top 25 aptamers selected as preferentially binding to Q78-huntingtin had a high percentage of guanine nucleotides in their sequences (Figure S2). Seventeen of these were selected and re-synthesized with a 5′ biotin-moiety. In an ELISA assay format, all 17 exhibited a significantly higher level of binding to mutant huntingtin, compared with normal huntingtin (Figure 1B; Table S1). The top four, named MS1, MS2, MS3, and MS4, were chosen for further characterization.

The presence of guanine-rich sequences in these Q78-huntingtin preferential binding aptamers suggested that they may share a guanine-rich DNA secondary structure, such as the G-quadruplex. Secondary structure analysis of the MS1, MS2, MS3, and MS4 aptamers

and control DNA oligonucleotides was performed with a thioflavin T (ThT) fluorescence assay[12,13] (Table S2). All four DNA aptamers exhibited strong fluorescence intensity signals, above background, in a concentration-dependent manner, consistent with the presence of G-quadruplexes (Figure 1C). Aptamer MS3 yielded the strongest signal, surpassing the positive control 45Ag oligonucleotide. This set of DNA aptamers that form intramolecular G-quadruplexes, therefore, highlights a secondary DNA structural motif that, by enabling preferential binding to Q78-huntingtin, can discriminate the protein with the elongated polyglutamine tract from Q23-huntingtin with the normal range polyglutamine segment.

Aptamers Bind the C-Terminal Arm of Q78-Huntingtin, near K2932/K2934 in CTD-II

To determine whether the MS1, MS2, MS3, and MS4 aptamers bind mutant huntingtin broadly or whether they may interact at a specific site or sites along the 350-kDa protein, we initially monitored the binding pattern using preformed complexes of biotinylated aptamers bound to Q78-huntingtin that had then been digested with the caspase-6 protease. This enzyme cleaves Q78-huntingtin (or Q23-huntingtin) at amino acid residue 586[14] (Figure 2A). This produced an ~80-kDa amino-terminal polypeptide bearing the polyglutamine stretch and a ~270 kDa C-terminal polypeptide regardless of aptamers binding (Figure S3). SDS-PAGE immunoblot analysis with anti-biotin antibody revealed that, although chemical crosslinking was not employed, the huntingtin-aptamer interaction was stable because the biotinylated MS1 aptamer signal was detected coincident with both the uncleaved Q78-huntingtin-aptamer complex (~350 kDa) and the 270-kDa C-terminal fragment, whereas the amino-terminal 80-kDa polypeptide, containing the polyglutamine segment, was not detected (Figure 2B). The same pattern was observed from cleaved complexes of Q78-huntingtin and biotinylated-MS2, -MS3, and -MS4 (Figure 2C). Immunoblot with huntingtin-specific antibodies HF1 (epitope residues 1,981–2,580) and mAb2166 (epitope residues 181–810) (Figures 2B and 2C) confirmed the expected identities of the cleavage polypeptides.

A subsequent analysis narrowed the interaction site of the strongest Q78-huntingtin binder, MS3, by surface lysine methylation (SLM) of preformed Q78-huntingtin-MS3 complex, followed by mass spectrometry (liquid chromatography-mass spectrometry [LC/MS/MS]), as judged relative to SLM of controls: Q78-huntingtin after interaction with a nonspecific aptamer GCdx, which showed little or no binding to either Q23- or Q78-huntingtin (Figure 2F) and Q78-huntingtin without aptamer. We also performed SLM with LC/MS/MS for the same set of Q23-huntingtin, such as bound with either MS3 or GCdx and no aptamer. Because SLM modifies exposed Lys or Arg residues,[15] the data were analyzed to identify the Lys and Arg residues whose methylation was hindered by MS3 binding, compared with modified peptides in control samples. The overall amounts of methylated peptides from all samples tested were comparable (Figure S4). First, we evaluated 49 and 54 Lys/Arg residues from methylated peptides (>2) in Q23- and Q78-huntingtin alone, respectively (Table S3). To quantify the differentials, we assigned a score for each modified site by considering the specificity and magnitude of change of the methylated peptides from MS3-bound Q23- or Q78-huntingtin compared with control GCdx aptamer-bound and unbound Q23- or Q78-huntingtin (equation is presented in Materials and Methods). Lysines K2932/K2934 had an outstanding score (~40) in Q78-huntingtin compared with other sites (<10), while showing a score close to zero in Q23-huntingtin (Figures 2D and 2E). These results strongly suggest that these two residues within the CTD-II domain of the C-terminal arm of Q78-huntingtin may be in the proximity of the MS3 aptamer binding site or may be directly involved in MS3 aptamer binding. To test this prediction, we replaced the CTD-II K2932/K2934 lysine residues of Q78-huntingtins with aspartic acid (D) residues (Figure 2F). For comparison, we evaluated binding to Q78-huntingtin with a substitution at another lysine residue within the C-terminal arm UCD domain: K2449D (Figure 2F), a site exhibiting the second highest SLM score (Figure 2E; Table S3). We also tested a triple-mutant Q78-huntingtin K2449D/K2932D/K2934D with both the UCD and CTD-II substitutions (Figure 2F). Consistent with the SLM result of MS3 binding in the proximity of the K2932/K2934 residues, the Q78-huntingtin K2932D/K2934D double mutation nearly eliminated the interaction with MS3, which showed the preferential affinity for the non-mutated Q78-huntingtin compared with non-mutated Q23-huntingtin, whereas the K2449D mutant showed slightly reduced affinity (Figure 2F). Notably, although decreased compared with unmutated Q78-huntingtin, biotinylated MS3 exhibited a stronger interaction with the K2449D/K2932D/K2934D triple mutant Q78-huntingtin, relative to the K2932D/K2934D double mutant Q78-huntingtin. This implies that the MS3 interaction likely entails access that can be modulated by features formed by the neighboring UCD and CTD-II domains in the context of Q78-huntingtin with its elongated polyglutamine segment in NTD-I. Therefore, our finding strongly suggests that the polyglutamine expansion at the very amino-terminal region globally alters the secondary and/or tertiary structure of mutant huntingtin, resulting in the formation of preferential binding sites near its end of the C-terminal region for aptamers.

Aptamer Binding Abrogates the Heightened PRC2-Stimulating Activity of Q78-Huntingtin

In addition to its physical impact on mutant huntingtin structure, the elongated polyglutamine tract also endows a gain of function. We monitor one such activity, heightened stimulation of PRC2 activity, that provides a valuable biochemical assay with which to conduct huntingtin structure-function studies. This abnormal activity of mutant huntingtin emerged from studies of normal huntingtin's essential role as a facilitator of the chromatin regulator during embryonic development.[9] Normal huntingtin stimulates PRC2-mediated deposition of the histone H3K27me3 mark in cells and, with purified recombinant proteins, in cell-free PRC2 nucleosome-array histone H3K27me3 assay. However, mutant huntingtins exceed this level of stimulation in a manner that increases with the length of the expanded polyglutamine tract.[9,16] It is not yet known how this effect is achieved. However, we have observed

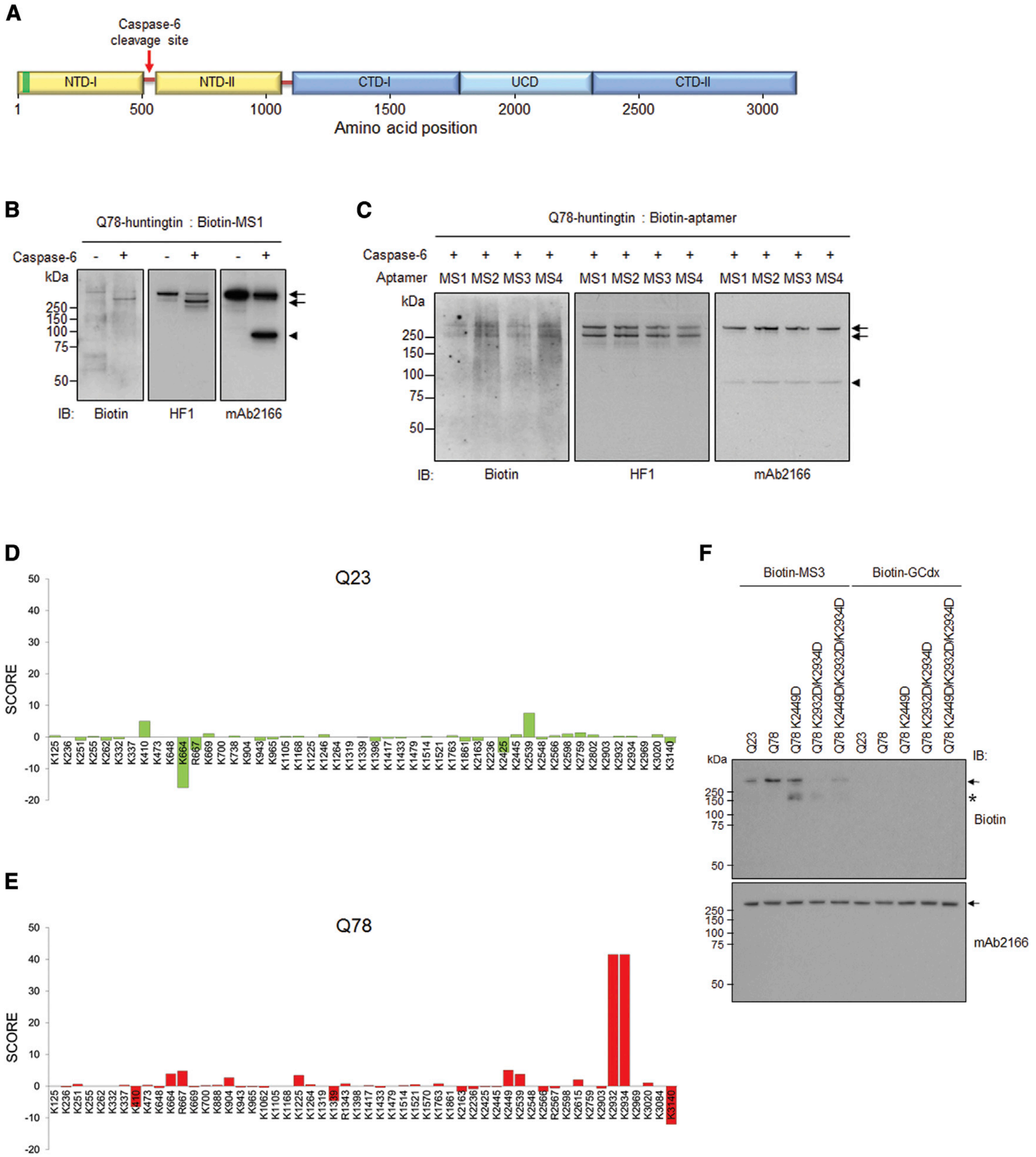

Figure 2. CTD-II Domain near K2932/K2934 in Mutant Huntingtin Is a Potential Binding Site of the MS3 Aptamer

(A) A schematic view of huntingtin with five domains delineated as previously described.[8] Caspase-6 cleavage site is indicated by the red arrow. The polyglutamine tract is denoted by the green bar. (B) The biotinylated DNA aptamer MS1 (Biotin, first blot) was detected in uncleaved Q78-huntingtin, and the larger fragment from caspase-6 cleavage of Q78-huntingtin with an anti-biotin antibody. The representative blot showed that most of the aptamer appeared to be bound to the C-terminal fragment and uncleaved huntingtin (arrows), but not the amino-terminal fragment (arrowhead), which was confirmed by re-probing the blot with a huntingtin C-terminal antibody (HF1, second blot) and an amino-terminal antibody (mAb2166, third blot). The experiment was repeated three times. (C) The same experiment was performed for the remaining biotinylated aptamers (MS2, MS3, MS4) and MS1. The representative immunoblot demonstrated that all aptamers preferentially bound to the C terminus of Q78-huntingtin. The experiment was repeated three times. (D and E) SLM analysis was performed with the huntingtin-aptamer complex and huntingtin alone. The methylated lysine residues were detected by LC/MS/MS and assigned a score based on the equation described in the Materials and Methods. The bar graphs showing the score of Q23- (D) and Q78- (E) huntingtin revealed that K2932/K2934 in CTD-II was the most probable binding site of MS3 on Q78-huntingtin. (F) Representative immunoblot showing purified Q23-, Q78-, Q78 K2449D-, Q78 K2932D/K2934D-, and Q78 K2449D/K2932D/K2934D-huntingtin bound with biotinylated MS3 or GCdx aptamers by probing with an anti-biotin antibody (Biotin) and an anti-huntingtin antibody (mAb2166). The location of full-length huntingtin is indicated by the arrow in both immunoblots. The asterisk marked a blurry band detected by anti-biotin antibody, but not by mAb2166, implying that a protein (or more than one) from insect cells, showing affinities to MS3 aptamer, was particularly enriched in Q78 K2449D-purified protein and less in Q78 K2932D/K2934D-purified proteins. The experiment was repeated three times.

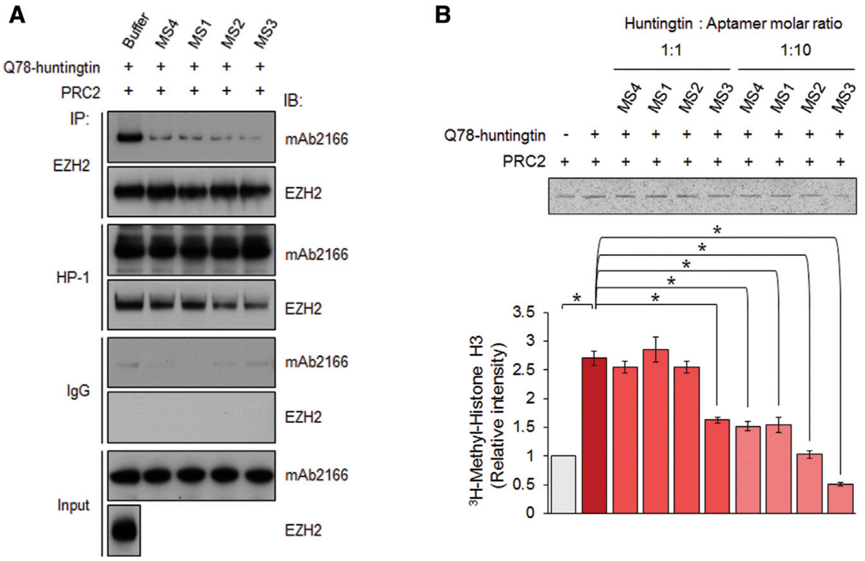

Figure 3. Aptamer Binding Selectively Reduces Mutant Huntingtin's PRC2-Stimulating Activity

(A) The affinities of Q78-huntingtin alone and huntingtin-aptamer complexes to PRC2 were compared by immunoprecipitation with an anti-EZH2 antibody and an anti-huntingtin antibody (HP-1), followed by immunoblotting for huntingtin and EZH2. The experiment was repeated three times. (B) Autoradiogram of bands of ^{3}H-methyl histone H3 produced by PRC2 in the absence and presence of mutant-huntingtin-preferring DNA aptamers. Bottom: the bar graph of the band densitometry results, showing that aptamer binding significantly reduced the ability of mutant huntingtin to enhance basal PRC2 activity. Mean band intensities were measured from three independent experiments. Asterisks indicate statistically significant differences compared with unbound Q78-huntingtin. Error bars represent SEM. *$p < 0.05$ (two-sided unpaired Student's t test).

that whereas normal huntingtin and mutant huntingtin both can interact (directly or indirectly) with PRC2 composed of EED, Rbp48, SUZ12, and EZH2, mutant huntingtin appears to interact more strongly, as judged by co-immunoprecipitation (data not shown and in preparation of this manuscript). We reasoned that because the G-quadruplex-forming aptamers preferentially bind to mutant huntingtin rather than normal huntingtin, the binding of these small molecules to the former may be expected to influence mutant huntingtin's exaggerated functional interaction with PRC2. Pre-binding of recombinant human Q78-huntingtin to DNA aptamers MS1, MS2, MS4, and especially MS3 decreased the interaction of mutant huntingtin with the PRC2 complex, as revealed by the reduced signals detected for huntingtin and EZH2 in co-immunoprecipitates, compared with the levels observed in the co-immunoprecipitates when unbound Q78-huntingtin was complexed with PRC2 (Figure 3A). The apparent decrease in the interaction with the complex implied that the aptamer binding may also attenuate Q78-huntingtin's heightened PRC2-stimulating activity. This hypothesis was tested using our previously reported cell-free reconstituted PRC2 nucleosome-array histone H3K27me3 assay.[9] At high concentrations (1:10 huntingtin/aptamer molar ratio), the MS1, MS2, MS3, and MS4 aptamer-bound recombinant human Q78-huntingtins all exhibited significantly decreased PRC2 stimulation, compared with unbound Q78-huntingtin. MS3 binding significantly decreased PRC2 stimulation even at a 1:1 aptamer-huntingtin ratio (Figure 3B). Thus, these aptamers, especially MS3, may significantly reduce mutant huntingtin's over-enhancement of PRC2 activity, perhaps by decreasing its affinity to PRC2.

Aptamer Binding Abrogates the Heightened PRC2-Stimulating Activity of Endogenous Mutant Huntingtin in Cultured HD-Derived NPCs

Endogenous mutant huntingtin is also associated with heightened stimulation of PRC2 in mouse *Hdh*Q111 CAG repeat knockin embryonic stem cells.[9,16] Moreover, the significance of PRC2 in adult neurons was reported, showing the critical role of PRC2-dependent transcriptional changes on progressive and fatal neurodegeneration in mice.[17] We therefore assessed whether the most functional DNA aptamer MS3 may discriminate the endogenous mutant and normal huntingtins expressed in previously reported[18] human HD60i4 and HD17m8 neuronal progenitor cells (NPCs), from an individual with the CAG expansion mutation and from a normal individual, respectively, and if so, whether the presence of cell-transfected aptamer may modulate PRC2 deposition of the histone H3K27me3 mark. Preferential interaction of MS3 with mutant huntingtin in transfected HD60i cells, compared with normal huntingtin in transfected HD17m8 cells, was supported by three lines of evidence. In a streptavidin-conjugated bead pull-down assay, the MS3-beads produced a 3.6-fold more efficient pull-down of huntingtin from HD60i4 lysate compared with HD17m8 lysate, although total huntingtin levels in the normal and mutant cells were similar (Figures 4A and 4B). In addition, whereas transfected FAM-conjugated GCdx control DNA oligonucleotide produced similar fluorescence intensities and puncta sizes in HD17m8 and HD60i4 NPCs, as detected by confocal microscopy (Figures S5A and S5C), transfection with FAM-conjugated MS3 aptamer yielded puncta of increased size and intensity in HD60i4 cells, compared with the HD17m8 cells (Figures S5A and S5B). Furthermore, analysis of co-immunostaining with an anti-huntingtin antibody (Figure 4C) and FAM-conjugated MS3 aptamer revealed a significantly increased co-localization of the signals in HD60i4, compared with HD17m8, neuronal cells (Figure 4D).

In order to monitor cellular PRC2 activity in the HD60i4 and HD17m8 cultured human NPCs, we first optimized an AlphaLISA H3K27me3 assay that could quantify the previously reported impact of the CAG expansion mutation in mouse embryonic stem cells and immortalized mouse striatal cells (see Materials and Methods;

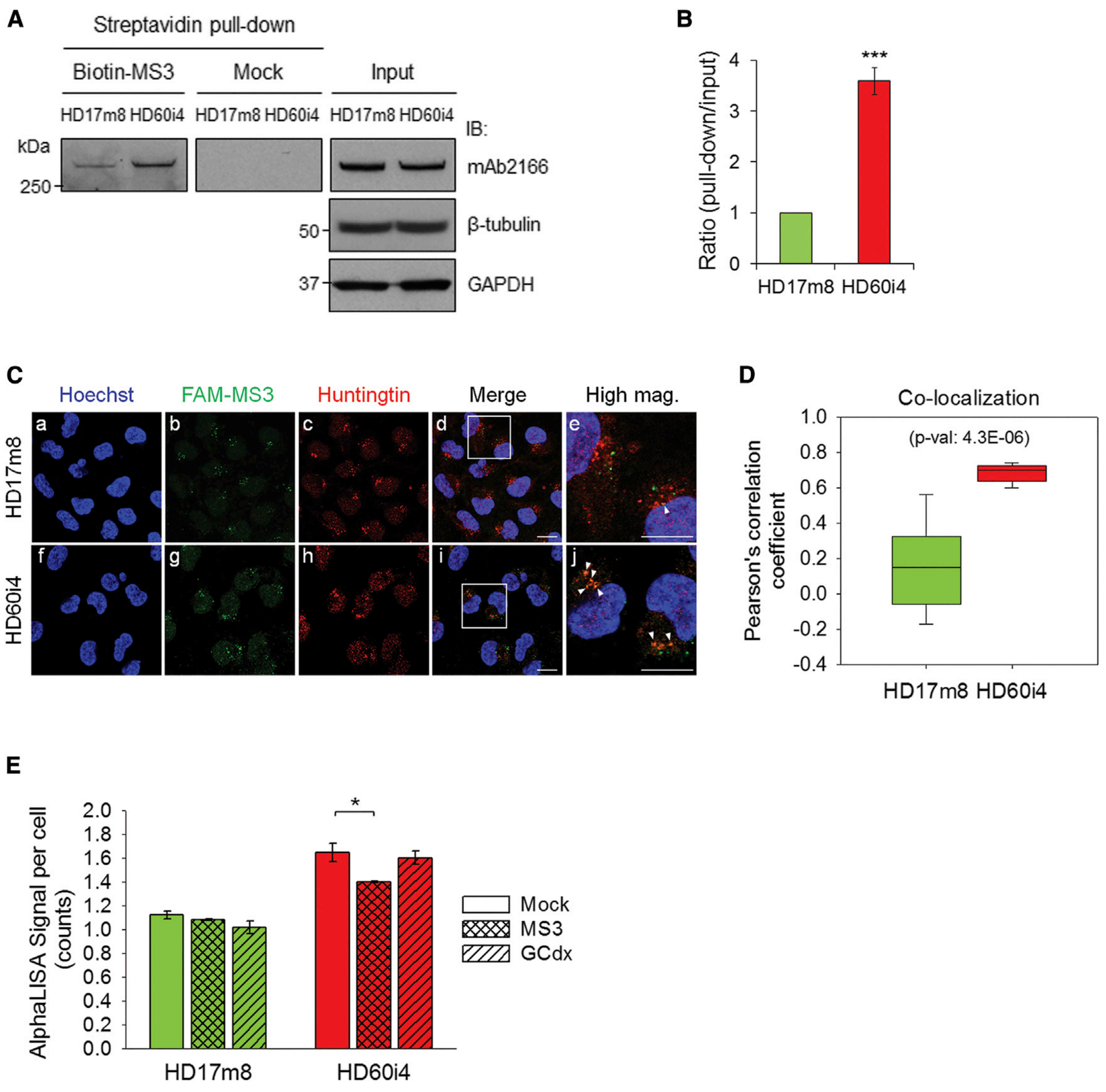

Figure 4. The MS3 Aptamer Can Bind to Endogenous Huntingtin and Abrogate Cellular PRC2 Activity

(A) Representative immunoblot showing streptavidin pull-down of huntingtin with or without biotinylated MS3 from the extracts of NPCs with CAG17/17 (HD17m8) and CAG60/18 (HD60i4) by probing with anti-huntingtin antibody (mAb2166). Huntingtin levels in the input are also shown (right). Both β-tubulin and GAPDH were used as loading controls. The experiment was repeated five times. (B) Densitometry analysis for streptavidin pull-down of endogenous huntingtin, normalized by huntingtin levels in the input that were normalized by β-tubulin. Mean band intensities were measured from three independent experiments. Error bars represent SEM. ***$p < 0.001$ (two-sided unpaired Student's t test). (C) Fluorescence images of HD17m8 and HD60i4 NPCs, showing the pattern of Hoechst 33342 (a and f) nuclei, transfected 6-FAM-conjugated MS3 (b and g), and anti-huntingtin (c and h) immunostaining, illustrating the significantly higher correlation between FAM-MS3 and cellular huntingtin in HD60i4 NPCs compared with HD17m8. The merged images (d and i) indicated by the open boxes are shown at a higher magnification in the adjacent columns (e and j). White arrowheads in (e) and (j) indicate FAM-MS3 aptamers that co-localized with cellular huntingtin. Scale bar, 10 μm. (D) Pearson's correlation coefficient for co-localization analysis of MS3-huntingtin signal overlap in HD17m8 and HD60i4 NPCs were displayed in a vertical boxplot. Mean degree of co-localization was measured in nine images per cell line. Error bars represent SEM. p value as determined by a two-sided unpaired Student's t test. (E) The cellular H3K27me3 levels measured by AlphaLISA signal were significantly decreased by MS3 aptamer transfection in HD60i4 NPCs specifically, but not by stem-loop DNA oligonucleotides (GCdx). Mean AlphaLISA signal counts were measured from three independent experiments. Error bars represent SEM. *$p < 0.05$ (one-way ANOVA with Tukey post hoc test).

Figure S6). This optimized AlphaLISA H3K27me3 assay detected increased signal/cell for mock-transfected HD60i4 NPCs, which express mutant huntingtin, compared with mock-transfected HD17m8 NPCs, which express only normal huntingtin. Furthermore, transfection of HD60i4 cells with MS3 aptamer yielded significantly decreased signal/cell, compared with mock transfection, while signal/cell was relatively unchanged in the MS3-transfected HD17m8 cells. Transfection with control aptamer GCdx mildly, although not significantly, decreased the AlphaLISA signal/cell in both the mutant and the normal NPCs (Figure 4E). Taken together, these findings in

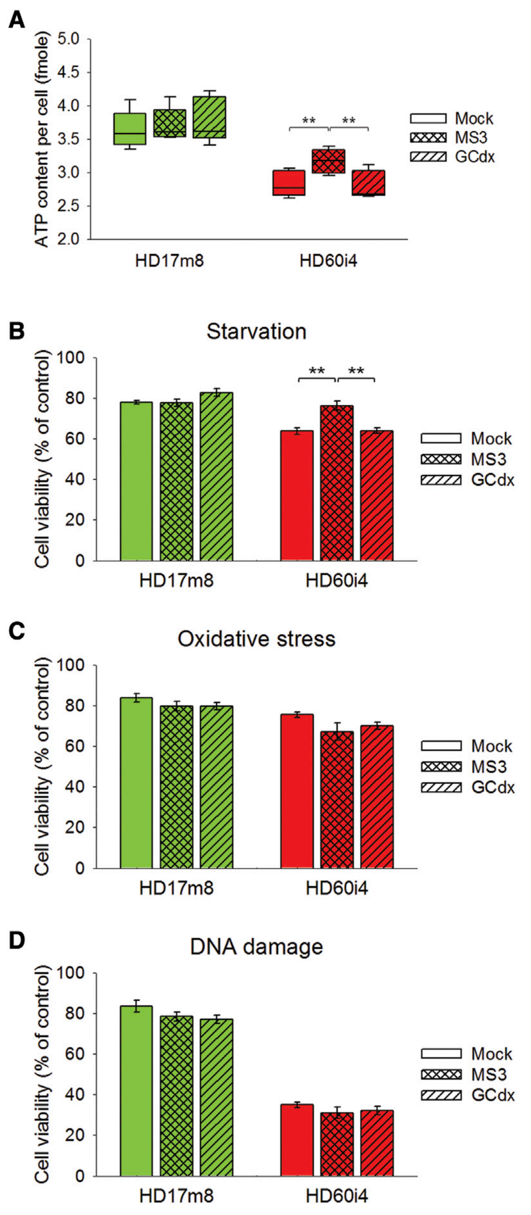

Figure 5. The MS3 Aptamer Protects HD Human NPC against Starvation-Dependent Stress

(A) The cellular ATP levels measured by bioluminescent assay (Promega) were significantly increased by MS3 aptamer transfection, but not by stem-loop DNA oligonucleotides (GCdx) in HD60i4 NPCs. Mean cellular ATP contents per cell were measured from four independent experiments. Error bars represent SEM. **$p < 0.01$ (one-way ANOVA with Tukey post hoc test). (B–D) The viability of HD60i4 NPCs starved in total starvation medium (HBSS) (B) for 6 hr was significantly increased by MS3 aptamer transfection, but not by GCdx. The viabilities of NPCs treated with 0.2 mM hydrogen peroxide (C) for 6 hr or 0.125 μM doxorubicin (D) for 24 hr were affected by neither MS3 nor GCdx transfection. Values are presented as the mean of three independent experiments. Error bars represent SEM. **$p < 0.01$ (one-way ANOVA with Tukey post hoc test).

transfected human HD NPCs support a preferential interaction of MS3 aptamer with endogenous mutant huntingtin that is associated with a diminution in the heightened stimulation of PRC2 that is one of the functional consequences of the impact of the elongated polyglutamine tract on the mutant protein.

Aptamer Binding Also Improves Energy Balance and Starvation-Dependent Stress Response in HD hNPCs, but Not in Normal hNPCs

The effect of MS3 on modulation of PRC2 activity prompted us to investigate whether DNA aptamer binding could possibly ameliorate mutant huntingtin toxicity. We have previously reported that the expanded polyglutamine repeats in huntingtin resulted in energetic/metabolic deficits in HD subjects as evidenced by decreased energy charge (ATP/ADP ratio) and ATP levels in human lymphoblastoid cell lines, mouse *Hdh* CAG knockin embryonic stem cells, and human induced pluripotent stem cells (iPSCs)/NPCs.[18–20] To determine the effect of MS3 on cellular ATP levels in human NPCs HD17m8 and HD60i4, the cells were mock transfected with MS3 or GCdx. As expected, ATP levels in HD60i4 cells were significantly lower than HD17m8. Interestingly, MS3 transfection significantly increased the ATP levels in HD60i4 cells compared with mock- and GCdx-transfected cells (Figure 5A), while there was no effect of MS3 or GCdx in HD17m8 cells. The improved energy homeostasis in HD human NPCs by MS3 prompted us to determine its impact on response of cells to various cellular stresses because huntingtin is reported to be involved in cell stress responses[21] that are largely associated with metabolic processes.[22] Compelling evidence suggests that increased oxidative stress and mitochondrial dysfunction may underlie HD pathogenesis.[23] Oxidative stress can result in DNA damage, and thus HD is associated with increased incidence of mutations. Interestingly, normal huntingtin has been reported to play a role in oxidative DNA damage repair, whereas mutant huntingtin is unable to facilitate it.[24] First, we compared the cell viabilities of normal and HD human NPCs under three different stresses: starvation, oxidative stress, and DNA damage. Interestingly, HD60i4 revealed significant reduction in cell viability under all three stress conditions compared with HD17m8 (Figure S7), indicating that mutant huntingtin could adversely affect these stress responses. In order to check the effect of MS3 on cell viability under these stress conditions, HD17m8 and HD60i4 were either mock, MS3, or GCdx transfected and subjected to each stress condition for an indicated time/concentration. While MS3-transfected HD60i4 showed significantly improved cell viability in response to starvation-dependent stress by almost achieving HD17m8 levels (Figure 5B), no effect on cell viability was observed under oxidative stress and DNA damage conditions (Figures 5C and 5D). Thus, MS3 aptamer binding to endogenous mutant huntingtin can ameliorate metabolic deficits of HD phenotype and enhance ability to combat starvation-mediated stress.

DISCUSSION

We have identified a specific set of DNA aptamers that preferentially bind to full-length huntingtin with an expanded polyglutamine tract, providing novel molecular tools that may modulate the structure and

function of the mutant protein, unlike the previously reported guanine-rich oligonucleotides that bind to the exon 1-encoded short amino-terminal huntingtin fragments inhibiting their aggregation.[25–27] In fact, two DNA aptamers (T30923 and T40216 as clone ID) of the previously identified guanine-rich oligonucleotides[25] were included in our 3416 DNA aptamers. Interestingly, they could not be shortlisted even as tightly bound aptamers (Figure S1), implying the unique specificity of our DNA aptamers toward full-length mutant huntingtin and the reliability of the microarray screening. However, it is not clear why all four WS (Q23-huntingtin-preferring) DNA aptamers could not be validated in the same ELISA assay showing low affinities to both Q23- and Q78-huntingtin. One of the possible reasons of exaggerated affinity toward Q23-huntingtin in the microarray may be because of Alexa 488 modification, which reacts with primary amines of huntingtin, on top of structural differences of Q23-huntingtin and Q78-huntingtin. We may need another screening strategy employing a crosslinking method to modify huntingtin or use unmodified huntingtin to screen Q23-huntingtin-specific DNA aptamers. In addition to the intrinsic benefits of aptamers (e.g., small size, easy chemical modification for labeling, and stability), the unique specificity of our aptamers to the altered secondary/tertiary structure of huntingtin as an outcome of posttranslational modifications or polyglutamine tract size may serve to support the development of aptamers as huntingtin biosensors. In particular, we clearly demonstrated the predominant binding of MS3 to endogenous mutant huntingtin in live human cells, indicating that the structural features of purified huntingtin precisely mirror endogenous huntingtin including the effects of polyglutamine expansion. The finding that the MS3 DNA aptamer interacts preferentially with Q78-huntingtin at a location at or near CTD-II K2932/K2934, perhaps involving UCD K2449, reasonably predicts that the preferential interactions of the other G-quadruplex-forming aptamers (MS1, MS2, and MS4) within the C-terminal arm of Q78-huntingtin are likely to involve the same features. It is noteworthy that the preferential binding of these DNA aptamers to Q78-huntingtin within the CTD-II domain, but not to Q23-huntingtin, is consistent with our previous report that the elongated polyglutamine tract of Q78-huntingtin subtly alters the physical structure of the entire mutant huntingtin molecule.[8] A single-molecule electron microscopy and chemical crosslinking-LC/MS/MS analysis show that Q23-huntingtin forms a spherical solenoid (with open cavity) in which the NTD-I domain of the amino-terminal arm is in close proximity to the CTD-I and CTD-II domains of the C-terminal arm. In Q78-huntingtin, the latter proximities of these domains with NTD-I are significantly altered,[8] implying a novel structural opportunity that would permit access to residues in the CTD-II domain to small molecules that preferentially bind Q78-huntingtin, such as our set of G-quadruplex-forming DNA aptamers.

G-quadruplex-forming molecules that can regulate biological processes through direct binding to specific proteins are emerging as promising therapeutic agents.[28–30] Indeed, all four aptamers tested decreased mutant huntingtin's enhancement of PRC2 activity in our cell-free PRC2 assay, and transfection of MS3 aptamer resulted in the reduced histone H3K27me3 mark only in HD NPCs expressing mutant huntingtin, measured by our bead-based PRC2 cellular assay. These findings support the general proposal that the HEAT/HEAT-like repeat domains of huntingtin can serve as a structural framework that interacts with PRC2 components, thereby facilitating the catalytic activity of the enzyme.[9] The detailed mechanism of huntingtin-PRC2 interaction is still elusive. However, binding of the DNA aptamers, which involves sites in the C-terminal CTD-II domain, is able to abrogate the heightened physical and functional interaction of Q78-huntingtin with PRC2, despite the presence of the elongated polyglutamine segment in the NTD-I domain. We also hypothesized that the aptamer binding could potentially contribute to relieve one or more HD phenotypic defects, such as energy deficit and hypervulnerability to cellular stresses. Indeed, MS3-transfected HD hNPCs revealed increased ATP levels and cell viability against starvation-induced stress. Because MS3 showed significantly increased affinity to endogenous Q62-huntingtin, it would be an interesting question to know whether MS3 can preferably bind to mutant huntingtin with shorter glutamine expansion, such as Q46-huntingtin. To directly test this, we performed the *in vitro* binding experiment with Q23-, Q46-, and Q78-huntingtin using biotinylated MS3 (Figure S8). Indeed, MS3 showed increased affinity to Q46-huntingtin, as well as Q78-huntingtin, compared with Q23-huntingtin. Thus, our DNA aptamers, such as MS3, can be developed to target mutant huntingtin with various polyglutamine lengths from adult-onset ranges of CAG expansion to juvenile-onset ranges.

We propose that the transfected MS3 aptamer may alter interaction of mutant huntingtin interaction with other protein complexes involved in metabolic regulation resulting in a protective effect without alteration in mutant huntingtin level in MS3-transfected human HD NPCs (Figure S5D). Based on these findings, it can be reasonably hypothesized that polyglutamine tract-length-dependent alteration of huntingtin secondary/tertiary structure may generate novel sites for protein-protein interaction, as well as abrogate certain interactions. These altered protein-protein interactions in mutant huntingtin may potentially result in its gain of function, and binding of aptamers at the novel interaction sites may revert the phenomenon, at least in part, by blocking or reducing these aberrant interactions. Extensive studies have identified numerous huntingtin binding partners involved in various cellular functions, but how those functions are affected by mutant huntingtin is largely unknown. Our aptamers can provide a novel way to understand the dynamics of protein complex interacted with mutant huntingtin in the absence or presence of metabolic stress.

Our findings, therefore, provide a proof-of-concept that abnormal activities conferred on mutant huntingtin by the expanded polyglutamine tract, encoded by the HD CAG expansion mutation, can be countermanded by delineating and exploiting the altered structural features of mutant huntingtin. The set of four G-quadruplex-forming DNA aptamers that target certain residues in the CTD-II, and possibly the UCD domain of the C-terminal arm of mutant huntingtin, discriminate mutant huntingtin from normal

huntingtin. These reagents now provide tools to determine the precise residues that are engaged, the structural changes in mutant huntingtin that are associated with dial-back of heightened function, a platform for generating mutant huntingtin-binding biosensors, and finally, the delineation of biological processes in cells and potentially in animal systems that are affected by mutant huntingtin. Also, considering huntingtin as a continuous α-helical solenoid, the detailed mechanism of MS3 binding to mutant huntingtin, including potentially significant structural changes caused by MS3 binding, should be pursued through independent studies, such as obtaining high-resolution images of MS3-bound mutant huntingtin structure, which will be important to develop our aptamer binding sites into therapeutic target sites for full-length mutant huntingtin.

MATERIALS AND METHODS

Full-Length Human Recombinant Huntingtin Expression and Purification

Expression of full-length Q23-, Q46-, and Q78-huntingtin from our pALHDQ23, pALHDQ46, and pALHDQ78 constructs was done using the Baculovirus Expression system (Invitrogen).[8] Three lysine aspartic acid (KD) mutant huntingtin constructs (K2449D, K2932D/K2934D, K2449D/K2932D/K2934D) were generated by submission of the DNA sequences to Genscript (Piscataway, NJ, USA), which provided synthesized DNA in the pFastBac1 vector using SalI/SacII restriction digest and standard molecular biology techniques. The preparation and purification of full-length huntingtin was carried out as previously described.[8]

Aptamer Screening Using the Recombinant Purified Huntingtin

To screen for small-molecule DNA aptamers that preferentially and specifically interact with huntingtin in a polyglutamine-dependent fashion, we used purified recombinant huntingtin as the bait. This screening was performed by using a protein-binding DNA aptamer microarray collection containing 3,416 aptamer probes in a single-strand DNA format (LC Science) with Alexa fluorophore 488 (Molecular Probes)-labeled Q23-huntingtin and Q78-huntingtin at the condition suggested by the manufacturer (Table S4). All raw data were processed and filtered for quality control, including background-subtracted and normalized signals and p values for the scatterplot of the image signal, to obtain statistically significant data. DNA aptamers that bound to huntingtin tightly were further selected by using a cutoff value of 50, which is the average of total aptamers' signal intensities. The DNA aptamers that preferentially bound to mutant huntingtin were further validated by ELISA using biotinylated DNA aptamers.

ELISA Assay

Huntingtin proteins (Q23 and Q78) (1 pmol/well) were coated in 96-well plates (Nunc MaxiSorp) in Tris-buffered saline (TBS) for 16–20 hr at room temperature. The wells were blocked with TBS buffer (10 mM Tris-HCl [pH 8.0], 0.15 M NaCl, 100 μL/well) containing 2 mg/mL BSA for 1 hr at room temperature. Indicated biotinylated aptamers (50 pmol) were added to the empty wells (controls) or wells coated with huntingtin and incubated for 2 hr at room temperature. After thoroughly washing with Tris-buffered saline with Tween 20 (TBST) (200 μL, 3×), diluted streptavidin-POD (1:10,000 dilution in TBST with 1% BSA, 100 μL/well; Roche) was added to all wells and incubated for 1 hr at room temperature. The streptavidin-POD solution was removed and washed thoroughly with TBST (200 μL, 3×). Peroxidase substrate (2,2′-azino-di-3-ethylbenzthiazoline-6-sulfonic acid and H_2O_2) was added and shaken until a blue-green color developed that was read at 405 nm. The intensity of color was proportional to biotinylated aptamer bound to huntingtin.

Thioflavin T Fluorescence Assay

DNA oligonucleotides (Integrated DNA Technologies) were heated at 90°C for 10 min in millipure water at 4, 20, or 40 μM. The oligonucleotides were diluted 1:2 in 100 mM Tris-HCl (pH 7.5), 100 mM KCl and slowly cooled to room temperature. Oligonucleotides were mixed with 1 μM thioflavin T (Sigma-Aldrich) and added to a 96-well assay plate to yield 1, 5, and 10 μM final concentrations of oligonucleotide and 0.5 μM final concentration of thioflavin T. Samples were mixed in triplicate. Fluorescence intensity was measured with a plate reader (Tecan Infinite M200) with excitation at 425 nm and emission at 490 nm.

Identification of Huntingtin Aptamer Binding Site

To identify whether the aptamers (MS1, MS2, MS3, and MS4) bound preferentially to the amino terminus of huntingtin bearing the polyglutamine region or to the C-terminal region of mutant huntingtin (Q78-huntingtin), we incubated biotinylated aptamers with Q78-huntingtin in a 10:1 molar ratio for 2 hr at room temperature followed by removal of free aptamers by subjecting the complex to spin gel-filtration columns (Sigma-Aldrich). Q78-huntingtin-aptamer complex was then subjected to caspase-6 digestion for 2 hr at 30°C followed by SDS-PAGE and western blotting. The nitrocellulose membrane was probed with anti-biotin antibody to detect biotinylated aptamer-bound huntingtin followed by stripping and re-probing with anti-huntingtin antibodies with epitopes in the amino- (mAb2166 from Millipore) and C-terminal (HF1) regions.

Surface Lysine Methylation Analysis with LC/MS/MS

The MS3 or GCdx aptamers were incubated with recombinant Q23- and Q78-huntingtin in a 5:1 molar ratio for 2 hr at room temperature. Huntingtin-aptamer complexes were mixed with a final concentration of 20 mM borane-dimethylamine complex (Sigma-Aldrich) and 40 mM formaldehyde (Sigma-Aldrich), and incubated 2 hr at 4°C. The reactions were further incubated with an additional final concentration of 10 mM borane-dimethylamine complex for 12 hr at 4°C. The reactions were quenched by adding a final concentration of 125 mM Tris-HCl (pH 8.0). The methylated huntingtin-aptamer complexes were separated on a 7.5% Tris-Glycine polyacrylamide gel (Bio-Rad), and the protein bands of interest were excised for LC/MS/MS analysis. Each sample was reduced with 1 mM DTT for 30 min at 60°C and then alkylated with 5 mM iodoacetamide for 15 min in the dark at room temperature. Gel pieces were then

subjected to a modified in-gel trypsin digestion procedure.[31] Gel pieces were washed and dehydrated with acetonitrile for 10 min followed by removal of acetonitrile. Pieces were then completely dried in a SpeedVac. Rehydration of the gel pieces was with 50 mM ammonium bicarbonate solution containing 12.5 ng/μL modified sequencing-grade trypsin (Promega) at 4°C. Samples were then placed in a 37°C room overnight. Peptides were later extracted by removing the ammonium bicarbonate solution, followed by one wash with a solution containing 50% acetonitrile and 1% formic acid. The extracts were then dried in a SpeedVac (~1 hr). Chymotrypsin (Roche Diagnostics) was then added to both the dried extracts and the residual gel pieces, and digested at room temperature overnight. The extraction of peptides was repeated and then dried. The samples were then stored at 4°C until analysis. On the day of analysis, the samples were reconstituted in 5–10 μL of high-performance liquid chromatography (HPLC) solvent A (2.5% acetonitrile, 0.1% formic acid). A nano-scale reverse-phase HPLC capillary column was created by packing 2.6 μm C18 of spherical silica beads into a fused silica capillary (100 μm inner diameter × ~30 cm length) with a flame-drawn tip.[32] After equilibrating the column, each sample was loaded via a Famos auto sampler (LC Packings) onto the column. A gradient was formed and peptides were eluted with increasing concentrations of solvent B (97.5% acetonitrile, 0.1% formic acid). As each peptide was eluted, they were subjected to electrospray ionization and then entered into an LTQ Orbitrap Velos Pro ion-trap mass spectrometer (Thermo Fisher Scientific). Eluting peptides were detected, isolated, and fragmented to produce a tandem mass spectrum of specific fragment ions for each peptide. Peptide sequences (and hence protein identity) were determined by matching protein or translated nucleotide databases with the acquired fragmentation pattern by the software program Sequest (Thermo Finnigan).[33] The modifications of 14.016, 28.0313, and 42.0470 mass units to lysine and arginine were included in the database searches to determine mono-, di-, and tri-methylation. All databases include a reversed version of all the sequences, and the data were filtered to 1%–2% peptide false discovery rate.[34] The amino acid numbering of methylated residues is based on the numbering of the reference cDNA (GenBank accession number L12392) for both Q23- and Q78-huntingtin.

The score for each methylated lysine residue was calculated based on the equation below:

$$|C - B| \times \left(1 - \frac{B}{A}\right),$$

where A is the number of MS-detected methylated peptides of huntingtin, B is the number of MS-detected methylated peptides of MS3 aptamer-bound huntingtin; and C is the number of MS-detected methylated peptides of GCdx aptamer-bound huntingtin.

Immunoprecipitation

To investigate the effect of aptamer binding on the physical interactions of huntingtin, pre-assembled human PRC2 complex and purified Q78-huntingtin protein were subjected to co-immunoprecipitation (IP). 2 μg of PRC2 complex and 2 μg of Q78-huntingtin protein pre-incubated with indicated DNA aptamers (MS1, MS2, MS3, or MS4) in 10-fold molar excess were incubated together for 1 hr at room temperature in IP buffer (50 mM Tris-HCl [pH 8.0], 150 mM NaCl, and 0.5 mM EDTA). 1 μg of anti-EZH2 antibody (BD Biosciences) or anti-huntingtin antibody (HP-1) was added subsequently and further incubated for 1 hr at room temperature. The antigen-antibody complex was pulled down by the addition of 50 μL of protein-A agarose slurry (Roche Applied Science) and incubated for 1 hr at room temperature. The immune complex was boiled in LDS sample buffer (NuPAGE) for 10 min. The eluted samples were separated by SDS-PAGE and subjected to immunoblotting with anti-huntingtin or anti-EZH2 antibodies.

Cell-free Assay for PRC2 Methyltransferase Activity

Purification of pre-assembled human PRC2 complex and preparation of the G5E4 nucleosomal array were carried out as previously described.[9] The reconstituted *in vitro* PRC2 activity assay was also performed similarly to the previous study[9] except for the following items: the reactions were done using 40 nM pre-assembled human PRC2 complex and purified Q78-huntingtin protein pre-incubated with indicated DNA aptamers after removing unbound aptamers using a spin column.

Cell Culture and Aptamers Transfection

To investigate cellular H3K27me3 level in various HD cellular models, we grew mouse embryonic stem cells (ESCs; $Hdh^{Q20/7}$ and $Hdh^{Q111/7}$), immortalized mouse striatal cells (ST$Hdh^{Q7/7}$ and ST$Hdh^{Q111/111}$), and human NPCs (HD17m8 and HD60i4) as previously described.[9,18,35] In brief, ESCs were maintained at 37°C on feeder layers of γ-irradiated mouse embryonic fibroblasts (Global Stem Sciences) or on gelatin-coated plates (1% gelatin solution; Millipore). Embryonic stem cell media contained Knock-Out D-MEM (Invitrogen), 15% FBS (Hyclone), 50 IU/mL penicillin, 50 mg/mL streptomycin (Invitrogen), 0.2 mM GlutaMAX (Invitrogen), 0.1 mM non-essential amino acids (Invitrogen), 0.1 mM 2-mercaptoethanol (Sigma-Aldrich), and 1000 U/mL leukemia inhibitory factor (LIF) (Millipore). The striatal cells were grown at 33°C in DMEM supplemented with 10% fetal bovine serum, 1% non-essential amino acids, 2 mM L-glutamine, and 400 mg/mL G418 (Geneticin; Invitrogen). Human NPC line HD17m8 was a generous gift of Dr. Steven J. Haggarty and was generated through directed differentiation of human iPSC GM08330 as described previously.[35] HD NPC line HD60i4 was generated from human iPSC HD60i4 as described previously.[18] HD60i-derived NPCs, expressing mutant huntingtin with a 62-glutamine tract, as well as normal huntingtin with a 20-glutamine segment, and HD17m8 NPCs from a normal individual, expressing huntingtins with a 19-glutamine segment have been described previously.[18] Cells were maintained on a poly-L-ornithine- and laminin-coated six-well plate (Falcon) at 37°C in neural expansion media (70% DMEM, 30% Hams F12, 1X B27 Supplement, 1% penicillin/ streptomycin, with 20 ng/ml fibroblast growth factor (FGF), 20 ng/ml epidermal growth factor (EGF), and 5 mg/ml heparin freshly added just before use). In order to stabilize single-stranded MS3 and GCdx DNA aptamers in cells, they were synthesized to include the five bases at both 5′ and 3′ ends

with 2′ O-methyl modification. Also, they included 6-FAM fluorescent dye at the 5′ end to be tracked in cells. Both NPC lines were transfected with indicated DNA aptamers using Viromer Black reagent (Lipocalyx), and all immunofluorescence experiments and bead-based cellular PRC2 activity assays were performed after 24 hr of incubation. The 6-FAM signal intensities of both MS3 and GCdx aptamers in NPCs were observed similarly after 48 hr of incubation (data not shown), indicating that they are stable in cells at least up to 48 hr.

Immunofluorescence

Human NPCs were grown on poly-L-ornithine- and laminin-coated coverslips for 24 hr, and after the experimental procedure, the cells were fixed with ice-cold methanol:acetone (1:1) for 5 min at −20°C, washed thrice, and permeabilized in 0.1% Triton X-100 in PBS for 5 min at room temperature. Cells were washed thrice with PBS and blocked with 10% FBS in PBS for 2 hr at 4°C. mAb2170 antibody (Millipore) was diluted as needed (1:250) in 10% FBS in PBS and incubated overnight at 4°C. Cells were washed again thrice with PBS and incubated with Alexa 555-conjugated secondary antibody (Molecular Probes) for 1 hr at room temperature and mounted in PermaFluor Aqueous Mountant (Lab Vision). Fluorescence images were captured at 63× magnification equipped with an SP5 AOBS scanning laser confocal microscope (Leica Microsystems).

Image Analysis

Identical image acquisition settings were used for like-stained samples. ImageJ/Fiji (coloc2 plugin and "analyze particle" module) were used for co-localization analysis and for measuring FAM-MS3 and FAM-GCdx puncta diameter. Nuclei were identified by DAPI staining (a threshold was applied and "analyze particles" was used to create a nuclei mask). For co-localization analysis of FAM-MS3 and huntingtin, the "subtract background" module was applied to each channel with a defined rolling ball radius, which was 50 for the FAM-MS3 channel and 40 for the huntingtin (mAb2170) channel. Background-subtracted channels, with the masks as guides, were then analyzed with the coloc2 plugin. Pearson's correlation coefficients were calculated with size exclusion, and threshold was applied to determine the relative degree of co-localization. For measuring the diameter of FAM-MS3 puncta, cell bodies were identified on the DAPI channel using a maximum filter with a radius of 140 to create a cell bodies mask, from which the identified nuclei were then subtracted to create a region of interest, "biomask." The "subtract background" module was applied to the FAM-MS3 and the FAM-GCdx channel with a rolling ball radius of 12. Then the "analyze particle" module was performed to measure the diameter of each FAM-MS3 and FAM-GCdx puncta, and the number of puncta possessing a diameter size above two pixels was then plotted.

Bead-Based Cellular PRC2 Activity Assay

The cellular assay for PRC2 was performed in accordance with the manufacturer's instruction (PerkinElmer). In brief, human NPCs were seeded on a poly-L-ornithine- and laminin-coated 96-well plate for 2 hr. Cells were transfected with indicated DNA aptamers using Viromer Black reagent (Lipocalyx) and incubated for 24 hr. After removal of the original culture medium, cell-histone lysis and extraction buffers were sequentially added to the cells. For AlphaLISA detection, histone extracts were transferred to wells of a 1/2 AreaPlate-96. The mixture of biotinylated anti-Histone H3 antibody and anti-H3K27me3 Acceptor beads in cell-histone detection buffer was added. Following a 60-min incubation at 23°C, streptavidin-conjugated donor beads were then added, and a final 30-min incubation was performed prior to plate reading with Enspire Alpha Reader (PerkinElmer). This optimized AlphaLISA H3K27me3 assay detected increased H3K27me3 signal/cell in heterozygous $Hdh^{Q111/7}$ CAG repeat knockin embryonic stem cells, expressing mutant huntingtin with 111 glutamines and wild-type mouse 7-glutamine huntingtin, compared with counterpart $Hdh^{Q20/7}$ *Htt* CAG knockin embryonic stem cells, expressing 20-glutamine huntingtin along with wild-type mouse huntingtin (Figure S6A), and yielded similar results for 111-glutamine huntingtin expressing ST$Hdh^{Q111/Q111}$ striatal cells, compared with their ST$Hdh^{Q7/Q7}$ wild-type huntingtin-expressing counterparts (Figure S6B), although with dramatically lower signal/cell, as expected for the developmental stage-appropriate drop in levels of the H3K27me3 mark.[16]

Cellular ATP Content Measurement

The ATP levels of human NPCs were measured by CellTiter-Glo Luminescent Assay kit (Promega). In brief, following the aptamer transfection and incubation period, the cells were incubated with a volume of CellTiter-Glo reagent equal to the volume of cell culture medium. Following 30-min incubation at room temperature, the ATP level of metabolically active cells was measured using a luminometer, and the experiments were repeated four times. ATP standard curve was generated by serial 10-fold dilutions of ATP disodium salt (Sigma-Aldrich) in culture medium (0, 1, 10, or 100 pmol). The ATP content per cell was determined with reference to ATP standards.

Cell Viability Assay under Various Stress Conditions

In order to compare the vulnerability between normal and HD human NPCs against three different stresses, starvation, oxidative stress and DNA damage, they were incubated with Hank's balanced salt solution (HBSS; Sigma-Aldrich),[36] culture medium containing hydrogen peroxide (H_2O_2; Sigma-Aldrich), or doxorubicin (Sigma-Aldrich), respectively, after transfection of Mock, MS3, or GCdx and subsequent 24-hr incubation. The cells were further incubated in 10-fold diluted PrestoBlue reagent (Invitrogen) containing culture medium for 1 hr at 37°C, and the absorbance values were measured at 570 and 600 nm using a microplate reader. The 570 nm values were normalized to the 600 nm values for the experimental wells and then corrected with the control values generated from the wells containing only culture media. The results were expressed as a percentage of untreated control cells (set at 100%).

SUPPLEMENTAL INFORMATION

Supplemental Information includes eight figures and four tables and can be found with this article online at https://doi.org/10.1016/j.omtn.2018.03.008.

AUTHOR CONTRIBUTIONS

B.S., R.V., M.E.M., and I.S.S. developed the concept. B.S., R.J., H.O., G.E.O., H.L., S.L.C., J.-M.L., J.-J.S., R.V., S.K., R.L., and I.S.S. designed the experiments and analyzed the data. B.S., R.J., and R.V. produced and purified proteins. R.V. performed aptamer screening using LC science platform and ELISA validation experiments, and R.V. and J.-M.L. analyzed the data. G.E.O. performed thioflavin T fluorescence assay. R.V. performed the aptamer binding experiment with caspase-6 cleavage. H.L. and J.-J.S. performed the aptamer binding experiment using SLM reaction, and B.S. analyzed the data. R.J. and B.S. validated MS aptamer binding sites with KD mutants. R.V. performed huntingtin interaction and cell-free activity experiments with PRC2. B.S. performed the binding experiment of MS3 with NPC lysates and bead-based PRC2 assay. B.S., H.O., and S.L.C. performed immunofluorescence experiments and analyzed the confocal image data. B.S., R.J., H.L., J.-J.S., R.V., and I.S.S. wrote the paper, which was significantly revised by S.K., R.L., and M.E.M.

CONFLICTS OF INTEREST

The authors declare no competing financial interests.

ACKNOWLEDGMENTS

We thank the members of the Cotman, Song, and Seong laboratories for suggestions and discussions. We are grateful to Dr. Ross Tomaino (the Taplin Mass spectrometry facility) for excellent technical support. This work was supported by the CHDI Foundation Inc. (J.-M.L., M.E.M., and I.S.S.), the NIH/National Institute of Neurological Disorders and Stroke (grant R01 NS079651 to I.S.S.), and a Global Research Laboratory grant from the National Foundation of Korea (grant NRF-2016K1A1A2912057 to I.S.S. and J.S.).

REFERENCES

1. The Huntington's Disease Collaborative Research Group (1993). A novel gene containing a trinucleotide repeat that is expanded and unstable on Huntington's disease chromosomes. Cell *72*, 971–983.

2. Snell, R.G., MacMillan, J.C., Cheadle, J.P., Fenton, I., Lazarou, L.P., Davies, P., MacDonald, M.E., Gusella, J.F., Harper, P.S., and Shaw, D.J. (1993). Relationship between trinucleotide repeat expansion and phenotypic variation in Huntington's disease. Nat. Genet. *4*, 393–397.

3. Lee, J.M., Ramos, E.M., Lee, J.H., Gillis, T., Mysore, J.S., Hayden, M.R., Warby, S.C., Morrison, P., Nance, M., Ross, C.A., et al.; PREDICT-HD study of the Huntington Study Group (HSG); REGISTRY study of the European Huntington's Disease Network; HD-MAPS Study Group; COHORT study of the HSG (2012). CAG repeat expansion in Huntington disease determines age at onset in a fully dominant fashion. Neurology *78*, 690–695.

4. Gusella, J.F., and MacDonald, M.E. (2000). Molecular genetics: unmasking polyglutamine triggers in neurodegenerative disease. Nat. Rev. Neurosci. *1*, 109–115.

5. Di Prospero, N.A., and Fischbeck, K.H. (2005). Therapeutics development for triplet repeat expansion diseases. Nat. Rev. Genet. *6*, 756–765.

6. Takano, H., and Gusella, J.F. (2002). The predominantly HEAT-like motif structure of huntingtin and its association and coincident nuclear entry with dorsal, an NF-kB/Rel/dorsal family transcription factor. BMC Neurosci. *3*, 15.

7. Andrade, M.A., and Bork, P. (1995). HEAT repeats in the Huntington's disease protein. Nat. Genet. *11*, 115–116.

8. Vijayvargia, R., Epand, R., Leitner, A., Jung, T.Y., Shin, B., Jung, R., Lloret, A., Singh Atwal, R., Lee, H., Lee, J.M., et al. (2016). Huntingtin's spherical solenoid structure enables polyglutamine tract-dependent modulation of its structure and function. eLife *5*, e11184.

9. Seong, I.S., Woda, J.M., Song, J.J., Lloret, A., Abeyrathne, P.D., Woo, C.J., Gregory, G., Lee, J.M., Wheeler, V.C., Walz, T., et al. (2010). Huntingtin facilitates polycomb repressive complex 2. Hum. Mol. Genet. *19*, 573–583.

10. Kaur, G., and Roy, I. (2008). Therapeutic applications of aptamers. Expert Opin. Investig. Drugs *17*, 43–60.

11. Keefe, A.D., Pai, S., and Ellington, A. (2010). Aptamers as therapeutics. Nat. Rev. Drug Discov. *9*, 537–550.

12. Gabelica, V., Maeda, R., Fujimoto, T., Yaku, H., Murashima, T., Sugimoto, N., and Miyoshi, D. (2013). Multiple and cooperative binding of fluorescence light-up probe thioflavin T with human telomere DNA G-quadruplex. Biochemistry *52*, 5620–5628.

13. Renaud de la Faverie, A., Guédin, A., Bedrat, A., Yatsunyk, L.A., and Mergny, J.L. (2014). Thioflavin T as a fluorescence light-up probe for G4 formation. Nucleic Acids Res. *42*, e65.

14. Wellington, C.L., Singaraja, R., Ellerby, L., Savill, J., Roy, S., Leavitt, B., Cattaneo, E., Hackam, A., Sharp, A., Thornberry, N., et al. (2000). Inhibiting caspase cleavage of huntingtin reduces toxicity and aggregate formation in neuronal and nonneuronal cells. J. Biol. Chem. *275*, 19831–19838.

15. Sledz, P., Zheng, H., Murzyn, K., Chruszcz, M., Zimmerman, M.D., Chordia, M.D., Joachimiak, A., and Minor, W. (2010). New surface contacts formed upon reductive lysine methylation: improving the probability of protein crystallization. Protein Sci. *19*, 1395–1404.

16. Biagioli, M., Ferrari, F., Mendenhall, E.M., Zhang, Y., Erdin, S., Vijayvargia, R., Vallabh, S.M., Solomos, N., Manavalan, P., Ragavendran, A., et al. (2015). Htt CAG repeat expansion confers pleiotropic gains of mutant huntingtin function in chromatin regulation. Hum. Mol. Genet. *24*, 2442–2457.

17. von Schimmelmann, M., Feinberg, P.A., Sullivan, J.M., Ku, S.M., Badimon, A., Duff, M.K., Wang, Z., Lachmann, A., Dewell, S., Ma'ayan, A., et al. (2016). Polycomb repressive complex 2 (PRC2) silences genes responsible for neurodegeneration. Nat. Neurosci. *19*, 1321–1330.

18. HDiPSC Consortium (2012). Induced pluripotent stem cells from patients with Huntington's disease show CAG-repeat-expansion-associated phenotypes. Cell Stem Cell *11*, 264–278.

19. Seong, I.S., Ivanova, E., Lee, J.M., Choo, Y.S., Fossale, E., Anderson, M., Gusella, J.F., Laramie, J.M., Myers, R.H., Lesort, M., and MacDonald, M.E. (2005). HD CAG repeat implicates a dominant property of huntingtin in mitochondrial energy metabolism. Hum. Mol. Genet. *14*, 2871–2880.

20. Jacobsen, J.C., Gregory, G.C., Woda, J.M., Thompson, M.N., Coser, K.R., Murthy, V., Kohane, I.S., Gusella, J.F., Seong, I.S., MacDonald, M.E., et al. (2011). HD CAG-correlated gene expression changes support a simple dominant gain of function. Hum. Mol. Genet. *20*, 2846–2860.

21. Nath, S., Munsie, L.N., and Truant, R. (2015). A huntingtin-mediated fast stress response halting endosomal trafficking is defective in Huntington's disease. Hum. Mol. Genet. *24*, 450–462.

22. Swan, C.L., and Sistonen, L. (2015). Cellular stress response cross talk maintains protein and energy homeostasis. EMBO J. *34*, 267–269.

23. Ayala-Peña, S. (2013). Role of oxidative DNA damage in mitochondrial dysfunction and Huntington's disease pathogenesis. Free Radic. Biol. Med. *62*, 102–110.

24. Maiuri, T., Mocle, A.J., Hung, C.L., Xia, J., van Roon-Mom, W.M., and Truant, R. (2017). Huntingtin is a scaffolding protein in the ATM oxidative DNA damage response complex. Hum. Mol. Genet. *26*, 395–406.

25. Skogen, M., Roth, J., Yerkes, S., Parekh-Olmedo, H., and Kmiec, E. (2006). Short G-rich oligonucleotides as a potential therapeutic for Huntington's Disease. BMC Neurosci. *7*, 65.

26. Yerkes, S., Vesenka, J., and Kmiec, E.B. (2010). A stable G-quartet binds to a huntingtin protein fragment containing expanded polyglutamine tracks. J. Neurosci. Res. *88*, 335–345.

27. Chaudhary, R.K., Patel, K.A., Patel, M.K., Joshi, R.H., and Roy, I. (2015). Inhibition of aggregation of mutant huntingtin by nucleic acid aptamers in vitro and in a yeast model of Huntington's disease. Mol. Ther. *23*, 1912–1926.

28. Brázda, V., Hároníková, L., Liao, J.C., and Fojta, M. (2014). DNA and RNA quadruplex-binding proteins. Int. J. Mol. Sci. *15*, 17493–17517.

29. Rhodes, D., and Lipps, H.J. (2015). G-quadruplexes and their regulatory roles in biology. Nucleic Acids Res. *43*, 8627–8637.

30. Gatto, B., Palumbo, M., and Sissi, C. (2009). Nucleic acid aptamers based on the G-quadruplex structure: therapeutic and diagnostic potential. Curr. Med. Chem. *16*, 1248–1265.

31. Shevchenko, A., Wilm, M., Vorm, O., and Mann, M. (1996). Mass spectrometric sequencing of proteins silver-stained polyacrylamide gels. Anal. Chem. *68*, 850–858.

32. Peng, J., and Gygi, S.P. (2001). Proteomics: the move to mixtures. J. Mass Spectrom. *36*, 1083–1091.

33. Eng, J.K., McCormack, A.L., and Yates, J.R. (1994). An approach to correlate tandem mass spectral data of peptides with amino acid sequences in a protein database. J. Am. Soc. Mass Spectrom. *5*, 976–989.

34. Beausoleil, S.A., Villén, J., Gerber, S.A., Rush, J., and Gygi, S.P. (2006). A probability-based approach for high-throughput protein phosphorylation analysis and site localization. Nat. Biotechnol. *24*, 1285–1292.

35. Sheridan, S.D., Theriault, K.M., Reis, S.A., Zhou, F., Madison, J.M., Daheron, L., Loring, J.F., and Haggarty, S.J. (2011). Epigenetic characterization of the FMR1 gene and aberrant neurodevelopment in human induced pluripotent stem cell models of fragile X syndrome. PLoS ONE *6*, e26203.

36. Shang, L., Chen, S., Du, F., Li, S., Zhao, L., and Wang, X. (2011). Nutrient starvation elicits an acute autophagic response mediated by Ulk1 dephosphorylation and its subsequent dissociation from AMPK. Proc. Natl. Acad. Sci. USA *108*, 4788–4793.

OMTN, Volume *11*

Supplemental Information

Novel DNA Aptamers that Bind to Mutant Huntingtin and Modify Its Activity

Baehyun Shin, Roy Jung, Hyejin Oh, Gwen E. Owens, Hyeongseok Lee, Seung Kwak, Ramee Lee, Susan L. Cotman, Jong-Min Lee, Marcy E. MacDonald, Ji-Joon Song, Ravi Vijayvargia, and Ihn Sik Seong

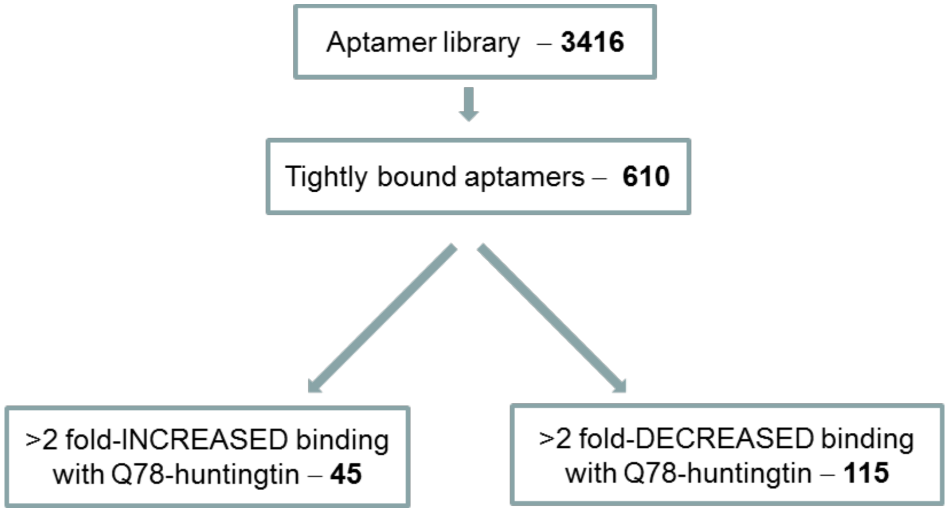

Figure S1. Experimental procedures.

Strategy for screening of Q78-huntingtin specific DNA aptamers.

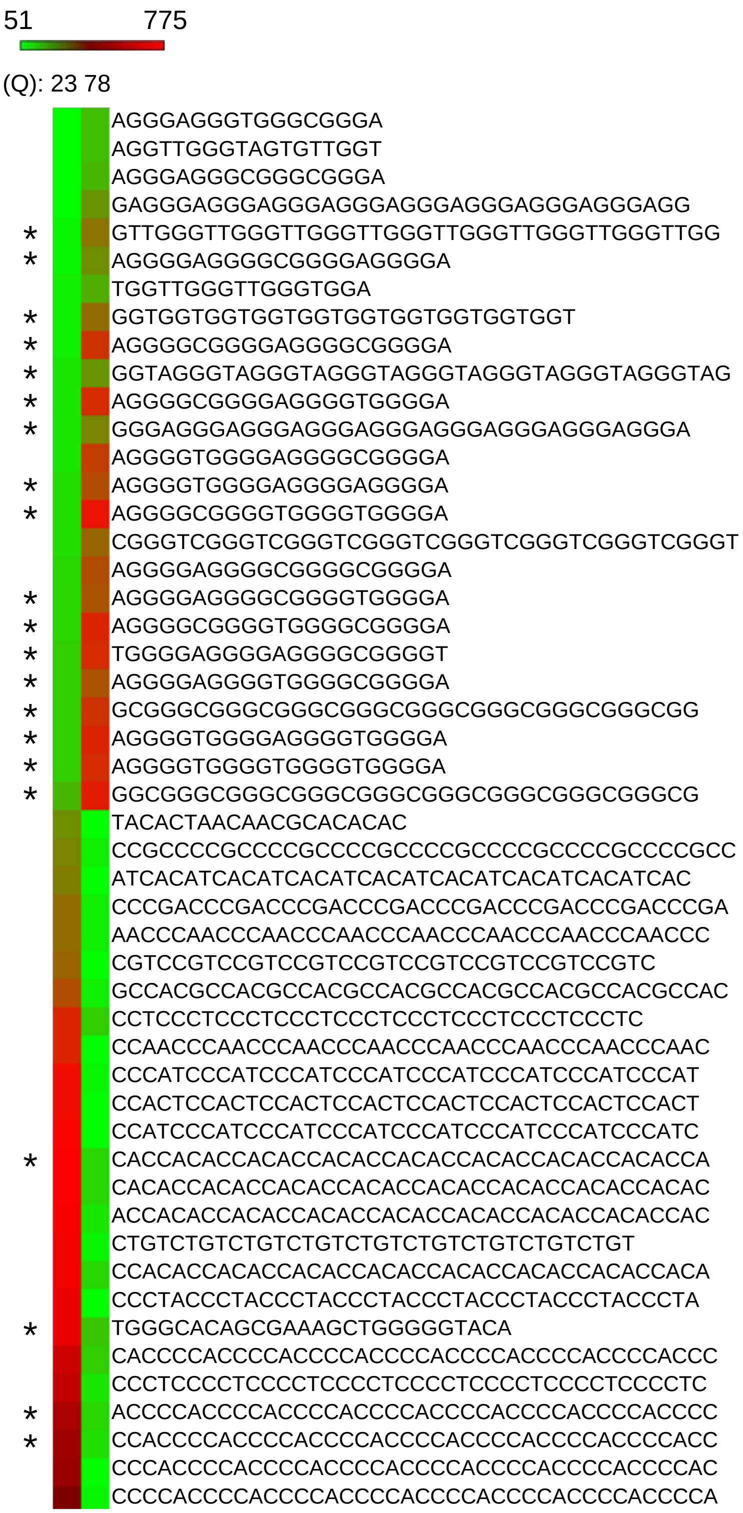

Figure S2. Selected DNA aptamers from microarray.

Heat map of Q23/Q78-huntingtin-preferring DNA aptamers and their sequences. High affinity is shown in shades of red while low affinity is shown in shades of green. Asterisks indicate 21 representative aptamers chosen for an ELISA assay.

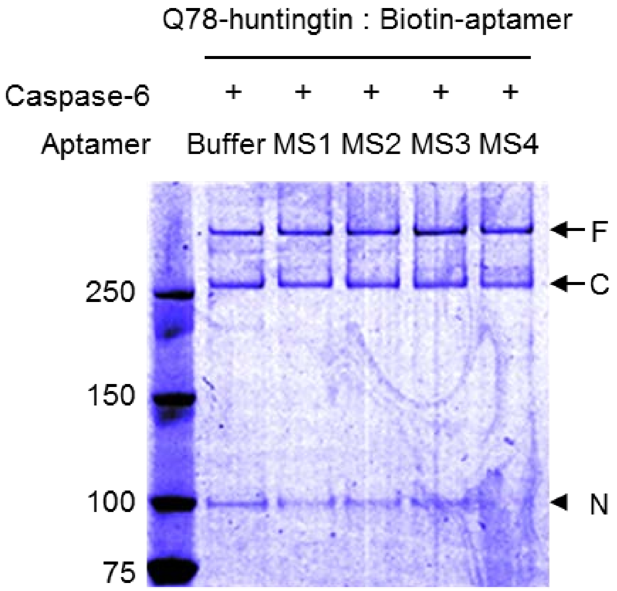

Figure S3. Aptamer binding does not alter cleavage of Q78-huntingtin by caspase-6.

Biotinylated DNA aptamer (MS1, 2, 3, 4)-bound Q78-huntingtin was cleaved by caspase-6. The cleavage products were separated by SDS-PAGE and detected by Coomassie staining. (F: full-length, C: carboxyl-terminal fragment, N: amino-terminal fragment)

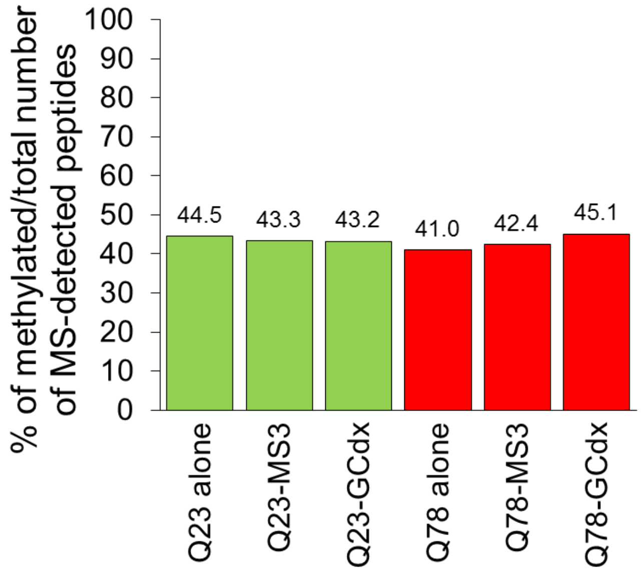

Figure S4. Identification of MS3 aptamer binding site.

Bar graph showing the percentage of methylated peptides within total MS-detected peptides from huntingtin alone or huntingtin-aptamer complexes indicating the overall methylation reaction of all samples was performed identically.

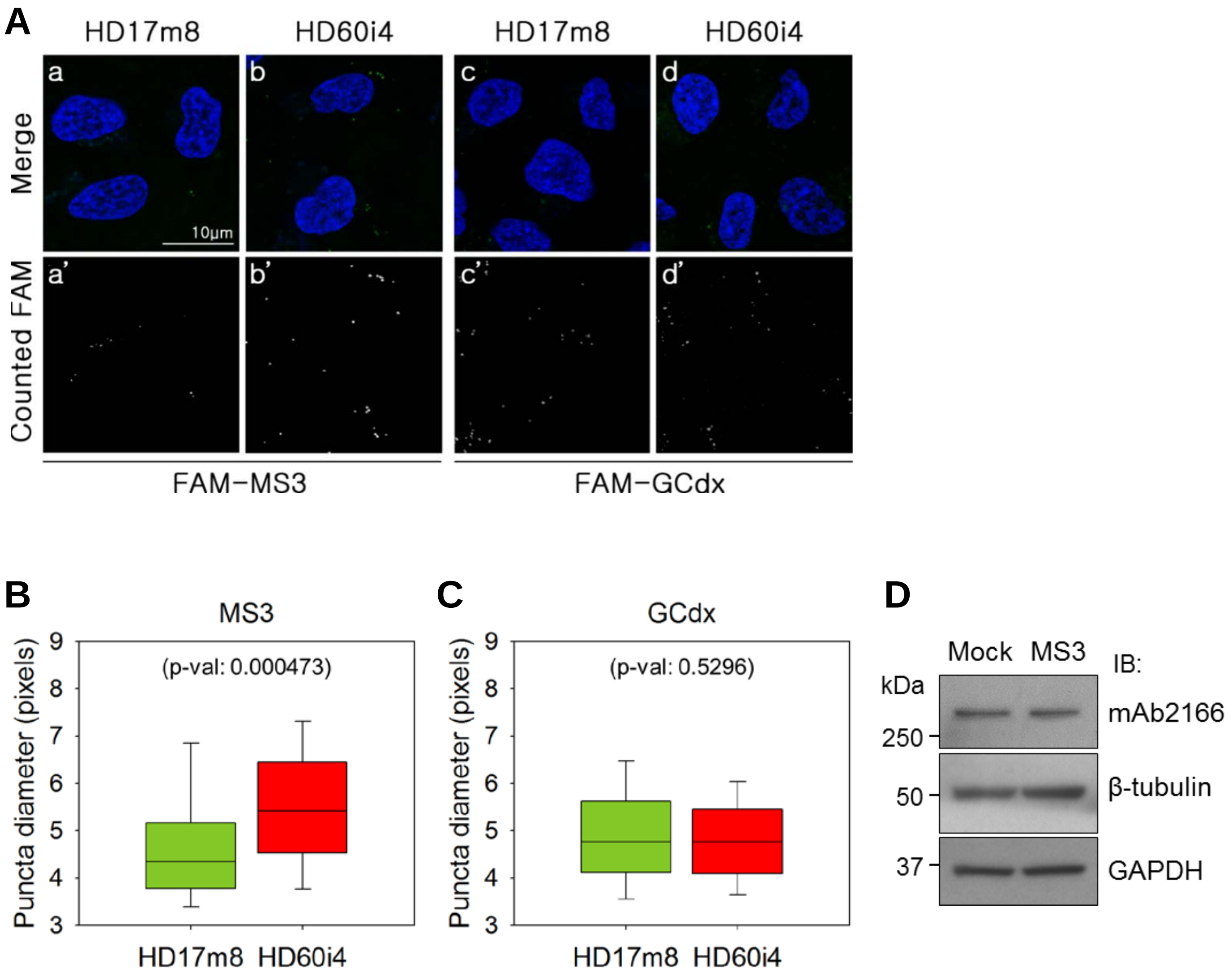

Figure S5. The accumulation of transfected FAM-MS3 aptamers was significantly increased in HD60i4 as compared to HD17m8.
(**A**) The merged fluorescence images of HD17m8 and HD60i4 NPCs (panel *a-d*), showing the Hoechst 33342 (blue) nuclei and transfected 6-FAM (green)-conjugated MS3 and GCdx signal, illustrating that the diameter of FAM-MS3 puncta were significantly enlarged in HD60i4 NPCs compared to HD17m8, whereas FAM-GCdx puncta showed similar fluorescence intensities in both HD17m8 and HD60i4 hNPC. Scale bar, 10 μm. (**B, C**) The diameter of each FAM-MS3 (B) and FAM-GCdx (C) puncta signal in HD17m8 and HD60i4 hNPC were displayed by vertical box plot. Mean puncta diameter was measured in 12 images per condition. Error bars represent S.E.M. *P*-values determined by a two-sided unpaired Student's *t*-test. (**D**) Immunoblot showing the amount of endogenous huntingtin without or with MS3 aptamer transfection in HD60i4 NPC by probing with anti-huntingtin antibody (mAb2166). β-tubulin and GAPDH, loading controls.

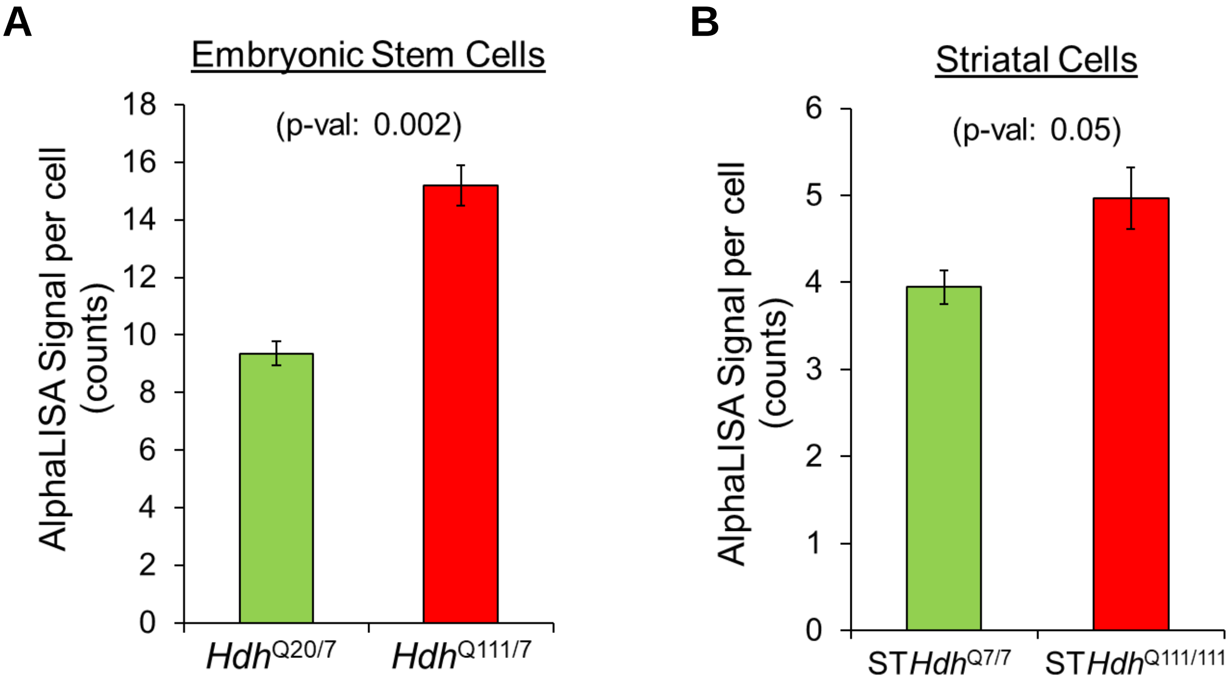

Figure S6. The endogenous levels for H3K27me3 in HD cellular models were significantly increased compared to wild-type.

(**A**) The AlphaLISA signal of H3K27me3 was significantly elevated in mouse embryonic stem cells with CAG 111/7 (*Hdh*$^{Q111/7}$) compare to cells with CAG 20/7 (*Hdh*$^{Q20/7}$). (**B**) The AlphaLISA signal of H3K27me3 was significantly elevated in *Hdh* CAG knock-in mouse striatal cell line with CAG 111/111 (ST*Hdh*$^{Q111/111}$) compare to cells with CAG 7/7 (ST*Hdh*$^{Q7/7}$). Mean of AlphaLISA signal was obtained from three independent experiments and the error bars represent S.E.M. *P*-values determined by a two-sided unpaired Student's *t*-test.

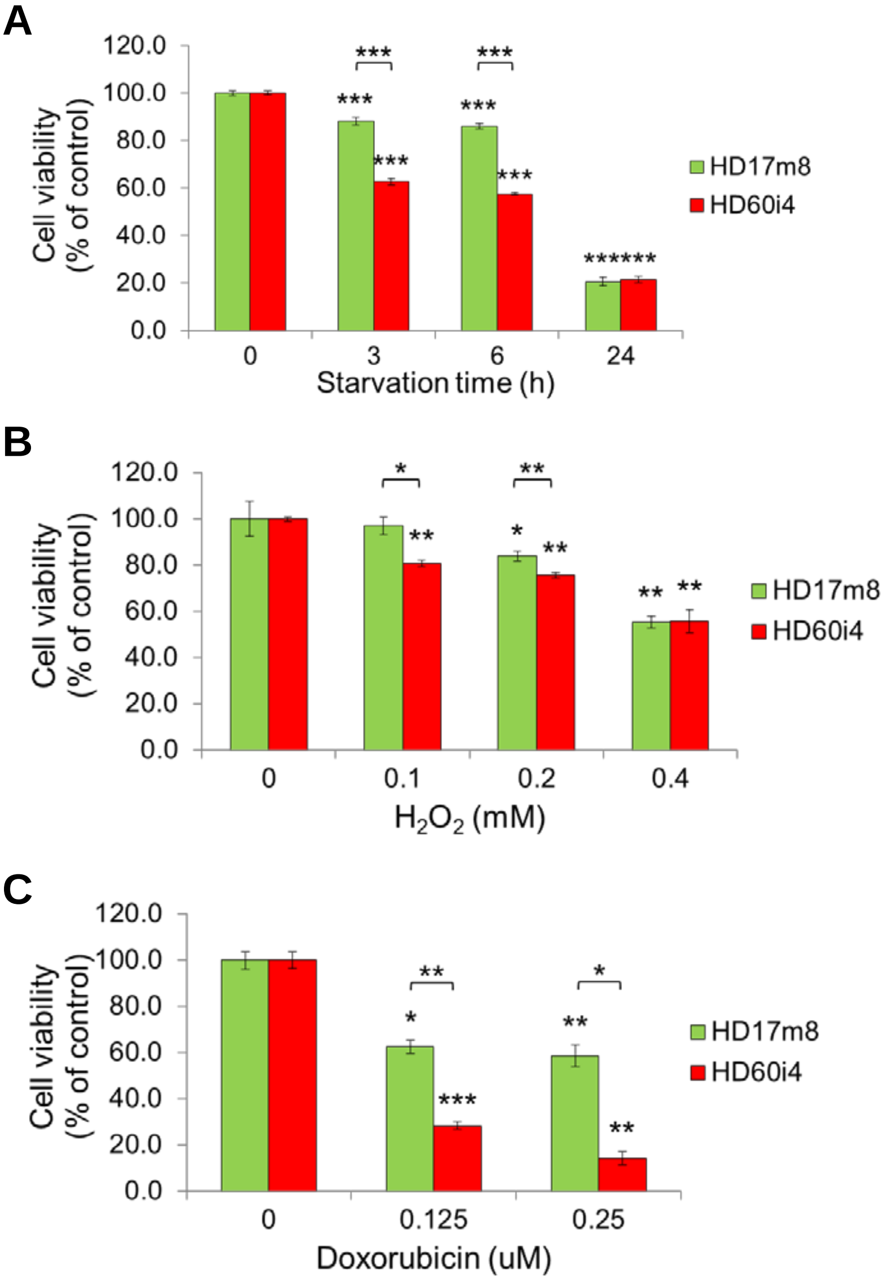

Figure S7. Measurement of cell viabilities in HD17m8 and HD60i4 NPCs under various cellular stress.

(**A**) Time course analyses of cell viability for NPCs starved in total starvation medium (HBSS) for 0, 3, 6 or 24 h. (**B**) Cell viability in NPCs cultured in the absence or presence of H_2O_2 (0.1, 0.2 or 0.4 mM) for 6 h. (**C**) Cell viability in NPCs cultured in the absence or presence of doxorubicin (0.125 or 0.25 μM) for 24 h. Values are presented as the mean of three independent experiments. Error bars represent S.E.M. *$P < 0.05$, **$P < 0.01$, ***$P < 0.001$ (two-sided unpaired Student's t-test).

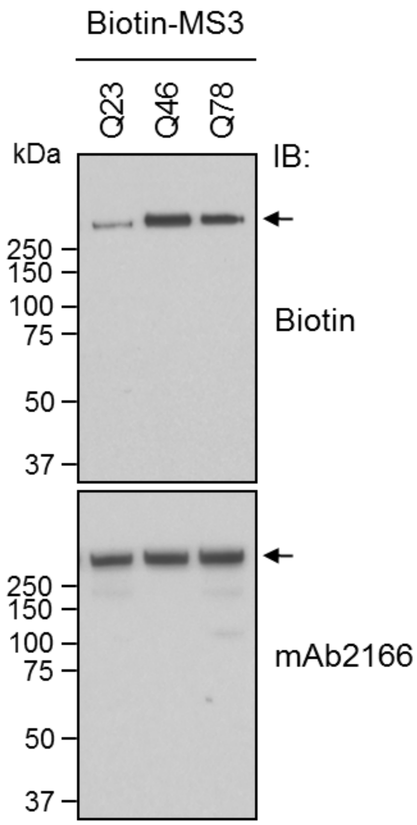

Figure S8. *In vitro* binding assay of MS3 aptamer with Q23-, Q46-, Q78-huntingtin. Representative immunoblot showing purified Q23-, Q46- and Q78-huntingtin bound with biotinylated MS3 aptamer by probing with an anti-biotin antibody (Biotin) and an anti-huntingtin antibody (mAb2166). The location of full-length huntingtin is indicated by the arrow in both immunoblots. The experiment was repeated three times.

Aptamer	DNA Sequence (5' to 3')	Microarray (Mean O.D.)		ELISA (Mean O.D.)	
		Q23	Q78	Q23	Q78
MS1	AGGGGTGGGGAGGGGTGGGGA	123.87	356.95	0.179	0.394
MS2	AGGGGTGGGGAGGGGAGGGGA	98.36	302.70	0.306	0.605
MS3	GGGAGGGAGGGAGGGAGGGAGGGAGGGAGGGAGGGA	88.53	227.67	0.347	0.730
MS4	TGGGGAGGGGAGGGGCGGGGT	118.40	363.94	0.152	0.388
MS5	AGGGGAGGGGCGGGGTGGGGA	109.69	295.69	0.228	0.327
MS6	AGGGGAGGGGTGGGGCGGGGA	122.09	296.94	0.078	0.241
MS7	AGGGGCGGGGAGGGGTGGGGA	87.54	347.76	0.115	0.262
MS8	AGGGGCGGGGTGGGGCGGGGA	110.22	354.84	0.142	0.244
MS9	AGGGGCGGGGAGGGGCGGGGA	83.37	333.58	0.066	0.184
MS10	AGGGGAGGGGCGGGGAGGGGA	71.63	211.30	0.125	0.174
MS11	AGGGGTGGGGTGGGGTGGGGA	125.42	348.68	0.031	0.069
MS12	GGTGGTGGTGGTGGTGGTGGTGGTGGT	82.49	260.67	0.103	0.185
MS13	GCGGGCGGGCGGGCGGGCGGGCGGGCGGGCGGGCGG	122.77	338.75	0.291	0.342
MS14	GGCGGGCGGGCGGGCGGGCGGGCGGGCGGGCGGGCG	155.39	366.39	0.303	0.298
MS15	GGTAGGGTAGGGTAGGGTAGGGTAGGGTAGGGTAGGGTAG	85.84	199.43	0.019	0.036
MS16	GTTGGGTTGGGTTGGGTTGGGTTGGGTTGGGTTGGGTTGG	64.26	252.61	0.140	0.316
MS17	AGGGGCGGGGTGGGGTGGGGA	99.25	378.31	0.244	0.363
WS1	CACCACACCACACCACACCACACCACACCACACCACACCA	423.64	118.00	0.095	0.159
WS2	TGGGCACAGCGAAAGCTGGGGGTACA	456.74	129.55	0.080	0.086
WS3	CCACCCCACCCCACCCCACCCCACCCCACCCCACCCCACC	552.47	123.68	0.039	0.065
WS4	ACCCCACCCCACCCCACCCCACCCCACCCCACCCCACCCC	651.73	110.78	0.048	0.120

Table S1. Table showing microarray and ELISA signal of the selected DNA aptamers with Q23/Q78-huntingtin. All 17 DNA aptamers that preferentially bound to Q78-huntingtin in the microarray experiment were consistent in ELISA assay while 4 DNA aptamers preferring Q23-huntingtin in the microarray experiment failed to be reproduced and, instead, showed low affinities to both Q23- and Q78-huntingtin in ELISA assay.

Aptamer	DNA Sequence (5' to 3')
MS1	AGGGGTGGGGAGGGGTGGGGA
MS2	AGGGGTGGGGAGGGGAGGGGA
MS3	GGGAGGGAGGGAGGGAGGGAGGGAGGGAGGGAGGGA
MS4	TGGGGAGGGGAGGGGCGGGGT
c-myc	TGAGGGTGGGTAGGGTGGGTAA
45Ag	GGGTTAGGGTTAGGGTTAGGGTTAGGGTTAGGGTTAGGGTTAGGG
ds26	CAATCGGATCGAATTCGATCCGATTG
GCdx	GCGCGCGCTTTTGCGCGCGC

Table S2. DNA sequences used for the Thioflavin T (ThT) fluorescence assay.

A

Position	Number of methylated peptides		
	Q23 alone	Q23-MS3	Q23-GCdx
K125	5	3	4
K236	5	5	8
K251	6	8	11
K255	5	4	5
K262	4	6	4
K332	10	12	9
K337	7	4	4
K410	8	6	26
K473	5	5	4
K648	10	10	8
K664	8	24	16
R667	7	11	4
K669	23	19	25
K700	13	13	17
K738	3	2	1
K904	3	6	6
K943	9	14	12
K965	6	10	9
K1105	5	4	5
K1168	7	6	4
K1225	37	37	32
K1246	4	1	0
K1264	9	10	10
K1319	5	5	5
K1339	19	20	18
K1398	4	5	10
K1417	79	88	84
K1433	48	56	54
K1479	4	5	5
K1514	10	8	7
K1521	12	10	10
K1763	5	3	2
K1861	18	22	16
K2163	7	11	13
K2236	6	6	8
K2425	5	11	7
K2445	4	3	6
K2539	34	18	34
K2548	6	10	11
K2566	7	6	3
K2598	3	2	5
K2759	9	5	8
K2802	3	1	0
K2903	4	4	5
K2932	46	34	35
K2934	46	34	35
K2969	5	4	4
K3020	11	10	2
K3140	76	80	45

B

Position	Number of methylated peptides		
	Q78 alone	Q78-MS3	Q78-GCdx
K125	4	4	6
K236	8	9	11
K251	9	7	10
K255	5	5	4
K262	6	6	6
K332	13	13	15
K337	6	5	7
K410	25	43	52
K473	6	4	5
K648	11	13	10
K664	40	14	20
R667	20	4	10
K669	16	20	21
K700	13	12	14
K888	3	2	3
K904	6	2	6
K943	15	17	15
K965	12	14	13
K1062	3	4	3
K1105	4	4	6
K1168	7	6	6
K1225	41	27	37
K1264	13	11	14
K1319	5	5	5
K1339	19	30	22
R1343	4	1	2
K1398	9	9	10
K1417	86	84	90
K1433	48	56	58
K1479	7	7	8
K1514	10	8	9
K1521	12	9	11
K1570	3	2	2
K1763	5	3	5
# K1861	5	31	46
K2163	7	9	15
K2236	5	9	10
K2425	7	8	9
K2445	7	8	9
K2449	6	0	5
K2539	20	17	42
K2548	10	8	8
K2566	3	8	7
R2567	5	6	3
K2598	3	3	3
K2615	5	0	2
K2759	6	6	9
K2903	3	4	6
K2932	52	12	66
K2934	52	12	66
K2969	4	4	5
K3020	3	2	5
K3084	3	3	3
K3140	35	45	87

Table S3. (**A, B**) Table showing the number of methylated peptides detected by LC/MS/MS from huntingtin alone or huntingtin (Q23/Q78)-aptamer complexes. Of note, there were a few sites (e.g., #K1861 in Q78-huntingtin) showing a highly negative score due to a high B/A (aptamer bound huntingtin/unbound huntingtin) value. This may have resulted from the incidental activation of the SLM reaction by the presence of aptamer on the surface near the site, suggested by the observation that, while GCdx exhibited no specific binding to huntingtin, methylation at that site in the presence of GCdx was relatively high compared to Q78 alone (46 versus 5 methylated peptides).

Category	Parameter	Description
Assay	Type of assay	*In vitro* Microarray chip
	Target	Human full-length huntingtin with Q23 and Q78 P42858 (HD_HUMAN)
	Primary measurement	Detection of fluorescence signal from huntingtin protein that bound tightly with single-strand DNA aptamer
	Key reagents	Alexa fluorophore labeled purified full-length huntingtin proteins
	Assay protocol	See the methods section titled in 'Aptamer screening using the recombinant purified full-length huntingtin'
	Additional comments	
Library	Library size	3,416
	Library composition	3,416 single-stranded DNA aptamer probes
	Source	Single-strand DNA format collected from literature or database
	Additional comments	
Screen	Format	Microarray
	Concentration(s) tested	1μM (200 μl)
	Plate controls	Multiple quality control probes are included on each chip
	Reagent/ compound dispensing system	Manual addition of proteins
	Detection instrument and software	Axon GenePix 4000B Microarray Scanner
	Assay validation/QC	Multiple quality control probes are included on each chip
	Correction factors	Background is calculated from the median of 5% to 25% of low intensity cells. BKG0 and blank cells are excluded for the background calculation
	Normalization	No Normalization
	Additional comments	
Post-HTS analysis	Hit criteria	Preferential binding to target Huntingtin proteins
	Additional assay(s)	17 screened DNA aptamers were further validated by ELISA assay
	Confirmation of hit purity and structure	17 Hits (DNA aptamers) were re-synthesized and their specificity to huntingtin was confirmed by ELISA
	Additional comments	

Table S4. High-throughput screening of DNA aptamers preferentially bind to mutant huntingtin.
